# Determination of stable carbon isotope ratios for molecules in natural organic matter using ESI FT-ICR MS

**DOI:** 10.1126/sciadv.aee5238

**Published:** 2026-06-17

**Authors:** Shuxian Gao, Boris P. Koch, Steffen Kümmel, Jan Tebben, Oliver J. Lechtenfeld

**Affiliations:** ^1^ Department Environmental Analytical Chemistry, Research Group BioGeoOmics, Helmholtz Centre for Environmental Research–UFZ, Permoserstr. 15, Leipzig D-04318, Germany.; ^2^ Alfred-Wegener-Institut, Helmholtz-Zentrum für Polar- und Meeresforschung, Am Handelshafen 12, Bremerhaven 27570, Germany.; ^3^ University of Applied Sciences Bremerhaven, An der Karlstadt 8, Bremerhaven 27568, Germany.; ^4^ Department Technical Biogeochemistry, Helmholtz Centre for Environmental Research–UFZ, Leipzig D-04318, Germany.

## Abstract

Fourier transform ion cyclotron resonance mass spectrometry (FT-ICR MS) enables nontargeted identification of molecular formulas in natural organic matter (NOM), yet molecular-level isotope analysis at natural abundance remains limited. Here, we present a comprehensive workflow for molecular formula–specific isotope analysis (MSIA) in NOM using FT-ICR MS, including calibration against universal reference materials (RMs). The central step of MSIA is an intensity-tuning strategy that, with sufficient spectral averaging, achieves sub–per mil (‰) precision and accuracy and enables robust isotopic comparison (Δδ^13^C) across samples. Between terrestrial and marine NOM, we observed Δδ^13^C values of 17.3, 8.8, and 10.1‰ for marine-enriched formulas C_19_H_22_O_10_, C_20_H_26_O_9_, and C_20_H_24_O_9_, respectively, whereas an invariant formula, C_18_H_22_O_6_, showed a nonsignificant difference (Δδ^13^C = 2.7‰, *P* = 0.296). Matrix effects for RMs spiked into NOM were within the method uncertainty, rendering existing RMs suitable for MSIA when matched in carbon number, peak intensity, and mass (<50 daltons). Under these conditions, molecular formula–level δ^13^C values were obtained, e.g., −46.8‰ for C_18_H_22_O_6_ in terrestrial NOM.

## Teaser

Formula-specific isotope analysis by FT-ICR MS reveals isotopic differences in natural organic matter across environments.

## INTRODUCTION

Variations in the relative abundance of stable isotopes in organic molecules arise from isotope fractionation and are expressed as subtle differences in isotope ratios (IRs), typically reported as per mil (‰) deviations relative to a standard. Stable isotope measurements of organic compounds provide insights into the ambient conditions of formation, source and biochemical processes, and forensic discrimination of their provenance (*1*).

IR mass spectrometry (IRMS), typically performed on multicollector magnetic sector instruments, is considered as the gold standard because of its high accuracy and precision. IRMS is commonly applied for bulk stable isotope analysis or compound-specific isotope analysis (CSIA). CSIA is often performed from hyphenated gas or liquid chromatography to target single compounds in mixtures. However, in highly complex mixtures such as natural organic matter (NOM), petrolum, or atmospheric aerosols, chromatographic separation of single compounds is not possible.

Ultrahigh resolution mass spectrometry (UHRMS), i.e., Fourier transform electrostatic trap mass spectrometry (Orbitrap) and Fourier transform ion cyclotron resonance mass spectrometry (FT-ICR MS), offers substantial potential for isotope analysis of molecular formulas (MFs) or even discrete molecules in natural samples. Recently, it has been shown that electrospray ionization (ESI) UHRMS achieves CSIA for both inorganic and organic molecules at natural abundances with sub-‰ precision and accuracy (*2*–*4*). However, advancements in IR analysis using UHRMS have so far been limited to purified analytes in matched matrices and are therefore not applicable to unknown molecules in NOM mixtures, which resist clean separation even by ultrahigh-performance liquid chromatography (*5*). Attempts to directly determine IRs of MFs within NOM matrices have demonstrated that FT-ICR MS can achieve measurement precision within the range of natural isotope fractionation (*6*). Nevertheless, the accuracy of these measurements remains insufficient for interpreting biogeochemical processes because substantial systematic errors in mass peak intensities compromise reliable IR comparisons. Consequently, the calibration of measured IRs to the conventional Vienna Pee Dee Belemnite (VPDB) scale with sub-‰ accuracy remains to be demonstrated for structurally uncharacterized MFs in NOM, for which no compound-specific reference materials (RMs) are available. 

Studying isotope distributions in NOM with UHRMS is complicated by multiple factors: (i) The substantial concentration variability of individual molecules across NOM samples challenges the robustness of IR measurements with UHRMS (*6*, *7*). (ii) The absence of RMs for molecules with unknown and variable isomeric compositions currently prevents the reliable conversion of isotopolog intensity ratios (Δ^13^C) to the conventional δ notation (δ^13^C) (*8*). However, isolating individual molecules from such complex matrices remains impractical (*2*). (iii) Matrix and ionization effects varying across target molecules and NOM samples may introduce additional uncertainty, but the extent is currently unknown. To overcome these challenges, key issues must be addressed before IR analysis using UHRMS can be applied to NOM:

1) Achieving robust IR comparisons for molecules present at varying concentrations across different matrices or NOM mixtures.

2) Establishing a universal calibration system for molecules lacking specific RMs. Similar to IRMS, this would involve correcting biases in UHRMS-measured IRs by referencing them to the measured IR of a standard.

In this study, we use FT-ICR MS for stable carbon isotope analysis of molecules in complex mixtures (here, NOM) and highlight key methodology steps and limitations. We show that targeting identical mass peak magnitudes of analytes across samples/matrices improves the precision and accuracy of measured IRs. The approach enables robust IR comparison (Δδ^13^C) for single compounds (i.e., RMs like caffeine) and for MFs in NOM. The influence of matrix effects from different sample types (marine versus terrestrial versus pure solvent) was evaluated using multiple RMs. By implementing a universal calibration system anchored to RMs that share the same number of carbon atoms with NOM formulas, the IRs of MFs (i.e., accurate molecular masses) in NOM can be converted and anchored to the conventional delta notion (δ^13^C). Given that the δ^13^C values for MFs in NOM represent the concentration-averaged δ^13^C ratios of all underlying structural isomers, we term this approach MF-specific isotope analysis (MSIA) to emphasize the difference to traditional CSIA. We applied our method to MFs indicative of marine NOM sources, confirming MF-level δ^13^C differences (Δδ^13^C) between marine and terrestrial samples. Using RMs and one-point calibration, we also estimated δ^13^C values for these NOM formulas, revealing substantial interformula variation in IRs.

## RESULTS AND DISCUSSION

### Workflow for stable carbon isotope measurements with FT-ICR MS

**Fig. 1.**
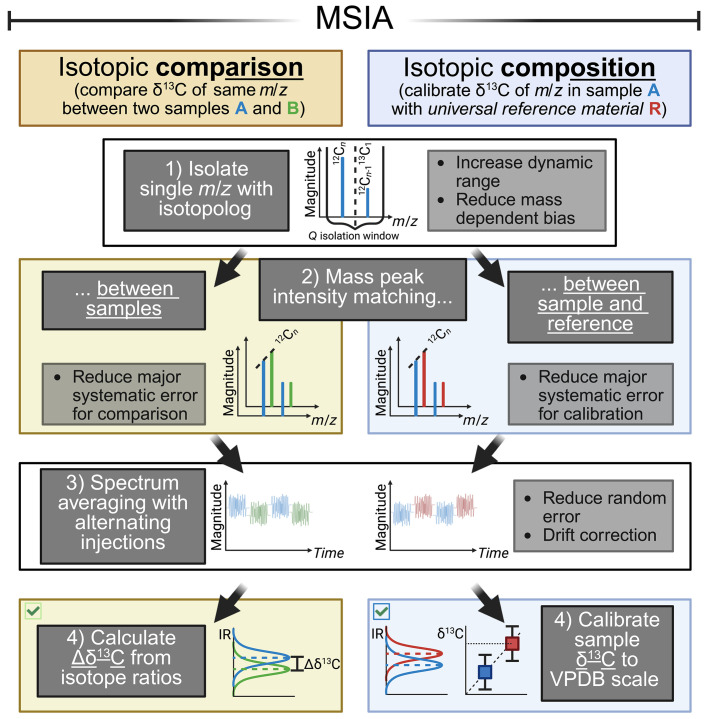
Implementation of MSIA for MFs (measured as *m*/*z*) in nonseparable complex mixtures like NOM. MSIA can be implemented in two different modes: Isotopic comparison for the same MF across two samples (**A** and **B**), yielding Δδ^13^C (i.e., differences in δ^13^C between samples; left branch), and determination of the isotopic composition of a distinct MF in sample **A** via normalization/calibration against universal RMs (**R**) with known δ^13^C_VPDB_ values, resulting in calibrated δ^13^C values (right branch). The approach is based on the following steps: (1) narrow *m*/*z* isolation to maximize ion intake into the analyzer, thereby improving ion sampling statistics and minimizing ion interference, space charge effects, and mass dependent bias; (2) intensity matching based on the monoisotopolog either via concentration adjustment and/or automatic gain control (AGC) or ion accumulation time (IAT) tuning to minimize the intensity dependent error on measured IRs; (3) spectral averaging to reduce the random error by increasing counting statistics (alternating injections help to compensate for instrumental drift over long measurement times); and (4) calculation of IRs and conversion to the δ^13^C scale. Steps (1) and (3) are fully equivalent for both modes, while steps (2) and (4) differ depending on whether the goal is isotopic comparison or calibration. Universal RMs refer to compounds with the same number of C atoms as analytes and closely matching *m*/*z* values (<50-Da difference) as they represent compounds with comparable SDs for ^12^C and ^13^C_1_ mass peaks. For an isotopic comparison, absolute δ^13^C values may deviate from those obtained from the isotopic composition mode, but the isotopic difference between two samples, expressed as Δδ^13^C, is reliable because of the applied intensity matching and matrix effects within analytical uncertainty. Created in BioRender. O. J. Lechtenfeld (2026), https://biorender.com/tqr5xrh.

Using a dual-inlet system, the terrestrial-derived Suwannee River fulvic acid (SRFA) standard and a surface seawater sample from the Weddell Sea, with dominant marine-sourced dissolved organic matter (DOM), were loaded into two syringes. An intensity tuning strategy was applied by adjusting sample concentrations to achieve comparable averaged mass peak magnitudes of the monoisotopologs at target mass/charge ratio (*m*/*z*) values across samples during measurements. A coefficient of variation (CV) below 2% was applied as the acceptance threshold for averaged mass peak magnitudes to achieve sub-‰ accuracy after calibration. The samples were interchangeably introduced into the FT-ICR MS, and the δ^13^C values of organic molecules present in different DOM samples were determined from the peak intensity of the ^12^C*_n_* monoisotopolog and the ^12^C_*n*−1_^13^C_1_ isotopolog. Thereafter, the stable carbon isotope variances, Δδ^13^C, of MFs were calculated and assessed (Fig. 1). The standard deviation (SD) and standard error (SE) of δ^13^C values across these consecutive spectra were calculated to assess the internal error and, hence, the internal precision.

### Isotope patterns for individual MFs in marine and terrestrial DOM

**Fig. 2.**
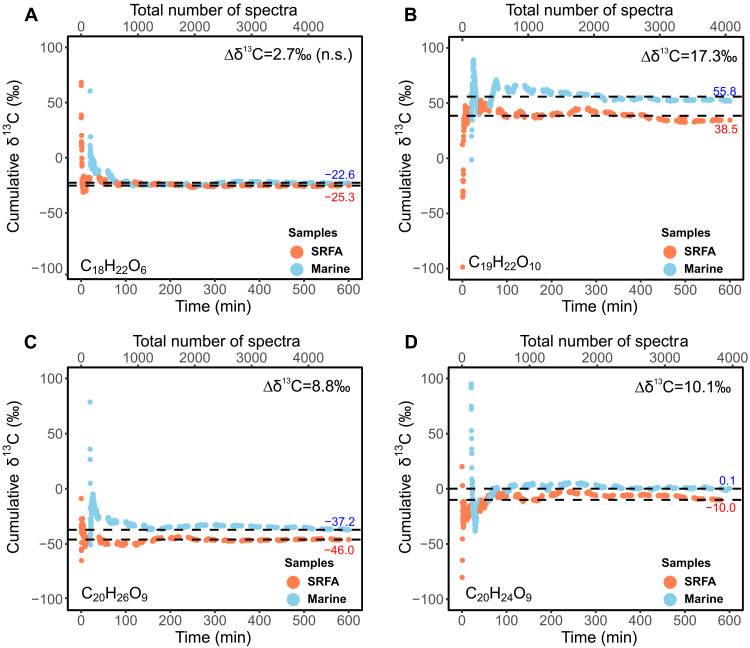
FT-ICR MS–derived δ13C values for individual MFs in terrestrial and marine DOM. Cumulative mean of δ^13^C values over time for four MFs in NOM, (**A**) C_18_H_22_O_6_ [*n* = 4467, averaged ^12^C*_n_* peak intensity: 1.06 ± 0.03 × 10^7^ (SD)], and three markers of marine inputs, (**B**) C_19_H_22_O_10_ [*n* = 4061, averaged ^12^C*_n_* peak intensity: 3.91 ± 0.11 × 10^6^ (SD)], (**C**) C_20_H_26_O_9_ [*n* = 4431, averaged ^12^C*_n_* peak intensity: 8.78 ± 0.36 × 10^6^ (SD)], and (**D**) C_20_H_24_O_9_ [*n* = 3955, averaged ^12^C*_n_* peak intensity: 3.82 ± 0.07 × 10^6^ (SD)]. n.s., not significant. Data are presented as the cumulative means of spectra acquired over time. The δ^13^C values were reported relative to VPDB (^13^C/^12^C = 1.11802%; δ^13^C = 0‰). Note that δ^13^C values are reported as raw instrument output, not as calibrated values, and that the position on the δ^13^C axis across (A) to (D) largely depends on the respective formula ^12^C*_n_* peak intensity (see the “Calibration strategy for NOM molecules without reference materials” section). Δδ^13^C values (on the top right) can be calculated from differences in δ^13^C values with comparable mass peak intensities after intensity tuning.

For the determination of the isotopic variance, or Δδ^13^C, four DOM MFs shared in terrestrial and marine samples were determined using the intensity tuning strategy (compare the “Ion population determines the accuracy and precision of δ^13^C measurement” section). The target formulas included one background MF common to both terrestrial and oceanic DOM without prominent environmental specificity (C_18_H_22_O_6_) and three biogeochemical markers that were previously shown to be relatively enriched in marine as compared to terrestrial DOM (C_19_H_22_O_10_, C_20_H_26_O_9_, and C_20_H_24_O_9_; Fig. 2). However, the correlations between mass peak abundance and the bulk δ^13^C values alone are insufficient to distinguish genuine isotopic variance from concentration differences across samples. Therefore, isotopic examinations are essential to further explore the biogeochemical significance and applicability of such DOM markers. For the MF C_18_H_22_O_6_, we did not detect significant isotope variance between terrestrial and marine samples (*P* = 0.296), with Δδ^13^C of 2.7 ± 2.5‰ (SE; Fig. 2A and fig. S19). One possible explanation is that this MF exhibited distinct δ^13^C values in the terrestrial and marine sources but converged through natural fractionation processes. Alternatively, the constant δ^13^C values across environments may indicate that C_18_H_22_O_6_ originated from terrestrial sources and resists biogeochemical processing before reaching the Weddell Sea surface. To validate this, structural information, e.g., via tandem mass spectrometry or at least polarity information from LC-FT-ICR MS (liquid chromatography–FT-ICR MS) analysis would be needed (*9*).

In agreement with the samples’ bulk δ^13^C values (table S1), all three marine markers displayed higher δ^13^C values in the marine sample than in SRFA with terrestrial origin, confirming that these markers were enriched in ^13^C in the marine samples (Fig. 2 and fig. S11), as expected from marine versus terrestrial primary production. The following isotopic differences (Δδ^13^C of the mean with SE) were determined for the marine markers: C_19_H_22_O_10_: 17.3 ± 4.7‰; C_20_H_26_O_9_: 8.8 ± 2.6‰; C_20_H_24_O_9_: 10.1 ± 4.1‰.

While bulk δ^13^C dissolved organic carbon (DOC) represents a concentration-weighted average isotopic pattern of all constituents, this application of MSIA indicates that carbon stable isotope patterns vary among individual MFs across samples. These results underscore both the intrinsic complexity of NOM and distinct isotopic responses of individual MFs to biogeochemical processes. Notably, given that the Δδ^13^C values of the three marine markers all exceeded the bulk δ^13^C difference of 4.5‰, other MFs present in both terrestrial and marine DOM likely exhibit only modest isotopic shifts as, e.g., observed for C_18_H_22_O_6_ or even negative shifts pointing to possible ^13^C enrichment in terrestrial samples (*10*).

### CSIA and MSIA in complex organic mixtures

#### Isotope variance for organic molecules is accessible with UHRMS

Recent studies indicated that the δ values of inorganic ions (δ^18^O, δ^15^N, and δ^34^S) and organic compounds (δ^13^C) can be precisely measured by Orbitrap MS (*2*, *11*). With sufficient effective ion counts (i.e., number of mass spectra), a precision of down to 1‰ (expressed as SE) can be achieved, comparable to conventional IRMS (*2*, *12*, *13*). This study achieved comparable results with 7 T FT-ICR MS with quadrupolar detection using caffeine standards (USGS63 and IAEA-600) measured with full mass range (table S1 and see the Caffeine_Int_differ dataset in Materials and Methods). To minimize concentration biases and matrix effects, caffeine standards were prepared at equal concentrations in ultrapure water. Two main ions, Na-monomer [M + Na]^+^ and Na-dimer [2M + Na]^+^, were formed under ESI-positive ionization mode (ESI+) (fig. S2). The measured ^12^C/^13^C ratios of USGS63 and IAEA-600 were 1.05 ± 0.09% (SD) and 1.03 ± 0.10% (SD) for the Na-monomer and 1.13 ± 0.12% (SD) and 1.11 ± 0.12% (SD) for the Na-dimer, respectively (fig. S3). Reported relative to VPDB, the δ^13^C values for USGS63 and IAEA-600 were −60.1 ± 2.1‰ (SE) and −79.1 ± 2.1‰ (SE) for the Na-monomer and 9.6 ± 1.8‰ (SE) and −4.8 ± 1.7‰ (SE) for the Na-dimer, corresponding to Δδ^13^C of 19.0 ± 3.0‰ (SE) and 14.4 ± 2.5‰ (SE), respectively (fig. S4). Given an inner precision of 3‰ per single measurement, achieving sub-‰ intermediate precision, expressed as the standard error of mean (SEM), requires a minimum of nine replicate measurements. Cumulative means of δ^13^C values initially fluctuated but stabilized after ~900 spectra per sample, demonstrating the potential for routine carbon isotope analysis of individual molecules (fig. S4).

#### Ion population determines the accuracy and precision of δ^13^C measurement

The calculated δ^13^C values systematically varied with the intensity of the ^12^C*_n_* monoisotopolog mass peak (*6*). In the full mass range measurements of caffeine, the Na-monomer appeared as the major peak under ESI+, accompanied by a Na-dimer at ~50% of the Na-monomer’s intensity (see the Caffeine_int_differ dataset in Materials and Methods and fig. S2). The δ^13^C values calculated form the Na-monomer and Na-dimer within the same measurement differed notably (fig. S4), indicating a systematic error of δ^13^C values related to variations in peak intensity, the number of carbon atoms, or the *m*/*z* range. The Na-dimer yielded δ^13^C values that aligned more closely with the expected reference values (−1.17‰ for USGS63 and −27.73‰ for IAEA-600) than those derived from the Na-monomer.

To evaluate the influence of peak intensity on measured δ^13^C values, caffeine and 17 other compounds (likely contaminants such as fatty acids) in the same mass spectrum of the caffeine standards were evaluated, each displaying a detectable ^12^C_*n*−1_^13^C_1_ isotopolog (table S3). Across all MFs, δ^13^C values ranged from −500 to 1000‰, reflecting a tendency toward positive offsets at lower monoisotopolog intensities [<10^6^ arbitrary units (A.U.); fig. S5, A and B]. As the intensity of the ^12^C*_n_* monoisotopolog increased 10-fold, measured δ^13^C values clustered within a progressively confined, symmetrical range around 0, eventually converging to within ±100‰ of the expected values (fig. S5, C and D).

The observed intensity-dependent variance in δ^13^C values resulted from statistical variability in ion sampling during ion intake (figs. S6 and S7) (*8*). Modern UHRMS instruments are capable of detecting analytes with less than 200 sampled ions while still yielding signal-to-noise ratios (SNRs) above the peak picking threshold of 4 (*14*). At low ion statistics (i.e., when fewer ions are detected), the absolute deviation from the theoretical ion count may be small. However, because the ion count is low, changes from 10^2^ to 10^5^ in ion counts can lead to large relative errors, resulting in high and positive shifts (~700‰) of the δ^13^C values (*8*). In addition, the SNR cutoff for peak picking suppress scans with weak ^12^C_*n*−1_^13^C_1_ peaks, leading to positively biased and unrepresentative averaged mean values (fig. S6, C and D). To ensure representativeness, the ^12^C_*n*−1_^13^C_1_ isotopolog mass peak intensity per carbon must exceed the SNR cutoff [4 in this study, corresponding to 10^4^ (A.U.)]. Although measurements with higher ion statistics show larger absolute errors, they improve the accuracy and precision of δ^13^C estimates because the variance is averaged over a larger total ion count.

In general, achieving high accuracy and precision in UHRMS-based IR measurements requires high and consistent sampled ion counts. In MSIA of complex mixtures like NOM, differences in analyte concentration and matrix effects across samples can affect and bias averaged ion counts and hence further affect the measured IRs. Assuming that ionization does not introduce isotope fractionation, reliable MSIA in NOM remains feasible despite ambient concentration biases and matrix effects, provided that the averaged mass peak magnitudes of the monoisotopologs are carefully matched across samples via intensity tuning strategies. This intensity tuning can be accomplished by adjusting analyte concentration, flow rate of injection, automatic gain control (AGC) targets (in Orbitrap instruments), or ion accumulation time (IAT; in FT-ICR instruments). Although AGC or IAT adjustments offer convenient means for tuning, the IR for a given molecule remains sensitive to time-dependent space charge effects that should be consistent across samples (*15*). These effects disproportionally affect low-abundance ions (i.e., the relevant isotopologs), causing them to decay more rapidly. This leads to an underrepresentation of heavier isotopes, ultimately introducing a negative offset to the measured δ^13^C values and thus resulting in biased IRs (*2*, *8*, *16*).

To test the effectiveness of the intensity tuning strategy, caffeine standards were remeasured (fig. S8 and the Caffeine_Int_match dataset in the Supplementary Materials). In this measurement, the averaged mass peak magnitudes of monoisotopologs were 1.74 ± 0.03 × 10^7^ with CV < 2% for the Na-monomer and 8.14 ± 0.81 × 10^6^ with CV < 10% for the Na-dimer. For the Na-monomer, δ^13^C values of 64.9 ± 1.9‰ (SE) for USGS63 and 40.8 ± 1.9‰ (SE) for IAEA-600 were obtained, resulting in a Δδ^13^C of 24.1 ± 2.7‰ (SE), closely resembling the expected 26.6‰. For the Na-dimer, measured δ^13^C values were 76.9 ± 2.0‰ (SE) for USGS63 and 54.4 ± 2.1‰ (SE) for IAEA-600, resulting in a Δδ^13^C of 22.5 ± 3.0‰ (SE), which is 4.0‰ lower than expected. Notably, the Na-monomer had lower variance in the averaged monoisotopolog intensities across samples and yielded more accurate Δδ values, underscoring the importance of intensity tuning strategies.

The performance of one-point and two-point calibrations was further evaluated with the Caffeine_Int_match dataset using a subset of the data from first 2500 spectra that were acquired (fig. S9). For the two-point calibration, USGS63 and IAEA-600 were measured for 20 min each as calibration standards for the USGS63 sample measured for the subsequent 20-min chunk (figs. S8 and S9A). This was repeated 14 times for each set of alternating USGS63 and IAEA-600. After just 677 spectra (equivalent to 2.7 hours), the signal intensity stabilized at 1.68 × 10^7^ and 7.64 × 10^6^ for the Na-monomer and Na-dimer, respectively, resulting in a δ^13^C accuracy of 1‰, which was achieved using the two-point calibration. For USGS63 (expected δ^13^C = −1.17‰), the calibrated δ^13^C values were −0.8 ± 4.4‰ (SE) for the Na-monomer and −1.7 ± 4.8‰ (SE) for the Na-dimer based on 677 spectra (figs. S8 and S9A). Higher throughput can be achieved at higher averaged mass peak magnitudes of ^12^C*_n_* monoisotopolog until space-charge effects become a limiting factor.

For one-point calibration, the IAEA-600 sample was used to calibrate the following USGS63 that would be measured for the next 20-min chunk (fig. S9B), again repeated 15 times throughout the complete run. For the Na-monomer, the one-point calibration yielded δ^13^C values of −3.7 ± 2.7‰ (SE) with all 2500 spectra for USGS63 (expected δ^13^C = −1.17‰) and an absolute error of 2.5‰ (figs. S8 and S9B). This systematic negative offset can be attributed to differences in averaged intensities of the monoisotopolog between samples. USGS63 exhibited a 2.4% higher averaged monoisotopolog intensity than IAEA-600, leading to the observed negative offset. Similarly, for the Na-dimer and on the basis of all 2500 spectra, USGS63 was calibrated to −5.2 ± 3.0‰ (SE) with an accuracy of 4.0‰. This larger error likely resulted from a substantial difference in the averaged monoisotopolog intensity, with USGS63 exhibiting a 14.1% higher average intensity than IAEA-600. The accuracy of the Na-dimer was still better than expected, primarily due to two main reasons: (i) The Na-dimer exhibited similar ion statistics to the Na-monomer. Although the Na-dimer was measured at about 50% of the intensity of the monoisotopolog, it contained twice as many carbon atoms, resulting in a comparable ^13^C_1_ isotopolog signal as compared to the Na-monomer and, hence, comparable accuracy and precision. (ii) Variations in the averaged mass peak magnitude, which led to inaccuracies in measured δ^13^C values, were averaged over a larger number of carbon atoms (i.e., 16 carbon atoms for the Na-dimer compared to 8 for the Na-monomer). This reduced the relative error per carbon, which is the basis for the reported δ^13^C values (see the “Calculation of δ^13^C from peak intensities in UHR mass spectra” in the Supplementary Materials) (*8*).

#### δ^13^C values are reproducible when monoisotopologs have similar mass peak magnitudes

**Fig. 3.**
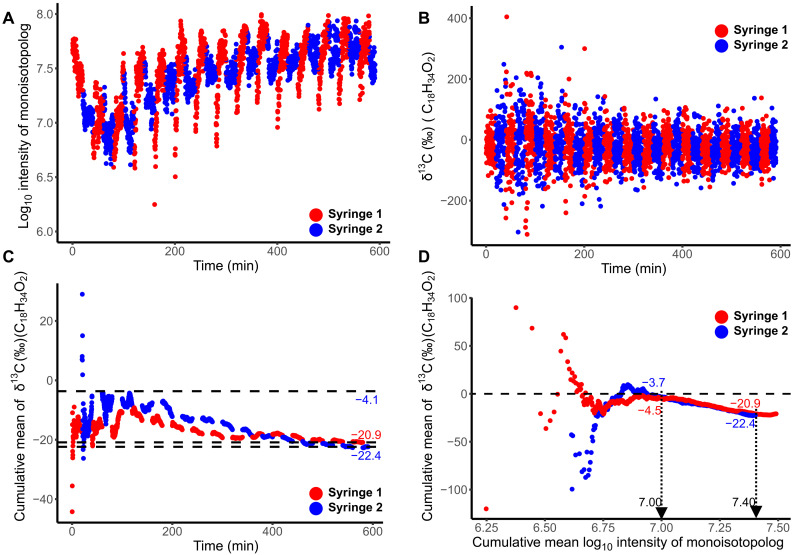
Impact of varying FT-ICR MS peak magnitude on measured IRs. Measured δ^13^C values for oleic acid (C_18_H_34_O_2_; δ^13^C is expected at −29.7‰) in ultrapure water with changing mass peak magnitude of the monoisotopologs (*n* = 1439 and 1402 in total for syringes 1 and 2, respectively): (**A**) Log_10_ intensity of the monoisotopolog over measurement time; (**B**) measured δ^13^C‰ values of oleic acid over measurement time; (**C**) cumulative mean of measured δ^13^C‰ values of oleic acid over the entire measurement time [the cumulative means of δ^13^C were −20.9 ± 1.6‰ (SE) and −22.4 ± 1.7‰ (SE) for syringes 1 and 2, respectively]; and (**D**) cumulative mean of measured δ^13^C‰ values of oleic acid with increasing log_10_ intensity of the monoisotopolog. When the averaged mass peak magnitude of the monoisotopolog reached comparable values, the cumulative means of δ^13^C were −4.5 ± 5.6‰ (SE, *n* = 287) and −3.7 ± 4.9‰ (SE, *n* = 324) at 10^7^ and −20.9 ± 1.9‰ (SE, *n* = 1138) and −22.4 ± 1.7‰ (SE, *n* = 1402) at 10^7.4^ for syringes 1 and 2, respectively. SEM values were 0.4 and 0.8‰ for these two averaged mass peak magnitudes. δ^13^C values are reported as raw instrument output, not as calibrated values (see the “Calibration strategy for NOM molecules without reference materials” section).

Concentration bias and ambient matrix effects can introduce asymmetry in ion populations (or mass peak magnitudes) of analytes in ion cyclotron resonance cells or Orbitraps, complicating CSIA and MSIA in NOM (*17*). Here, the effectiveness of intensity tuning was assessed across varying mass peak magnitudes of the monoisotopolog (see the Oleic_acid_Int_match dataset in Materials and Methods). Over the measurement time, the intensities of the monoisotopolog of oleic acid in both two syringes varied from 10^6.5^ to 10^8^ (A.U.), yielding δ^13^C values within −200 and +200‰ (Fig. 3, A and B). The cumulative mean of δ^13^C values of a single syringe declined by more than 18‰ over time as the intensities of the monoisotopolog increased by 10-fold, emphasizing the need of intensity tuning. The cumulative means were −20.9 ± 1.6‰ (SE) and −22.4 ± 1.7‰ (SE) for syringes 1 and 2, respectively (Fig. 3C). Note that for the reproducibility assessment, only raw instrument outputs were used rather than calibrated values (see the previous section and fig. S9). Instead of ordering the data over (measurement) time, δ^13^C values were cumulatively averaged according to increasing monoisotopolog mass peak magnitude (Fig. 3D). While variations in peak intensity can affect the raw δ^13^C accuracy, the relationship between the mass peak magnitude and the measured δ^13^C value was reproducible for two syringes (Fig. 3D and fig. S10). When the average mass peak magnitudes of the monoisotopologs were comparable at 1.00 ± 0.02 × 10^7^ (A.U.), the measured δ^13^C values showed excellent reproducibility with sub-% intermediate precision (SEM of 0.4‰) even with only ~300 spectra. This demonstrates the feasibility for comparatively rapid isotope analysis using UHRMS (45 min). Similarly, an SEM of 0.8‰ was obtained at average mass peak magnitudes of the monoisotopolog of 2.97 ± 0.03 × 10^7^ A.U. with CV < 1%.

Overall, an intensity tuning strategy can be used to assess the impact of ion population and to evaluate the feasibility of CSIA and MSIA for compounds in NOM. When average mass peak magnitudes of the monoisotopolog were comparable (CV < 2%), the SEM of repeated δ^13^C measurements remained below 1‰ for caffeine Na-dimers, maintaining sub-‰ precision, even under NOM matrix conditions, because of their doubled number of carbon atoms (figs. S12 and S14). The applied intensity tuning strategy is in accordance with reported best practices for conducting CSIA on Orbitrap instruments (*15*).

**Fig. 4.**
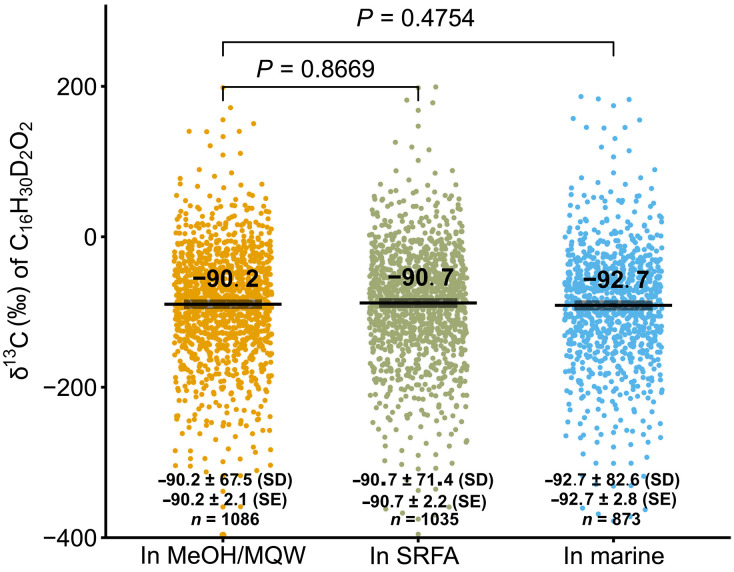
Matrix effects of δ^13^C measurements using FT-ICR MS. Palmitic acid-*d*_2_ was spiked into different matrices, and different concentrations were used for comparable mass peak magnitudes in mass spectra: 0.06 mg/liter in pure solvent (MeOH/MQW, 50:50, v/v), 0.1 mg/liter in SRFA (5 mg C/liter; 3S101F; MeOH/MQW, 50:50, v/v), and 0.15 mg/liter in marine DOM (10.9 mg C/liter; MeOH/MQW, 50:50, v/v), resulting in averaged mass peak magnitudes of the palmitic acid-*d*_2_ monoisotopolog of 2.81 × 10^7^ in solvent, 2.80 × 10^7^ in SRFA, and 2.77 × 10^7^ in marine DOM. The cumulative means of the δ^13^C values for palmitic acid-*d*_2_ were −90.2 ± 2.1‰ (SE, *n* = 1086) in pure solvent, −90.7 ± 2.2‰ (SE, *n* = 1035) in SRFA, and −92.7 ± 2.8‰ (SE, *n* = 873) in marine DOM. Samples were infused with an autosampler, and outliers with ^12^C*_n_* monoisotopolog peak intensities outside of 2 SDs of the averaged mean were excluded. On the basis of comparable averaged mass peak magnitudes of the monoisotopologs, an SEM of 0.8‰ was achieved for the δ^13^C analysis of the analyte across matrices. δ^13^C values are reported as raw instrument output, not as calibrated values (see the “Calibration strategy for NOM molecules without reference materials” section).

To further investigate potential matrix effects, we adjusted the concentrations of palmitic acid-*d*_2_ spiked into different NOM matrices to achieve comparable average ^12^C-monoisotopolog mass peak magnitudes with a CV below 1%. Robust δ^13^C values were obtained across matrices despite ion suppression effects (figs. S15 and S16). The uncalibrated raw δ^13^C values of palmitic acid-*d*_2_ were measured at −90.2 ± 2.1‰ (SE) in pure solvent [methanol/ultrapure water (MeOH/MQW)], −90.7 ± 2.2‰ (SE) in SRFA, and −92.7 ± 2.8‰ (SE) in the marine surface seawater DOM (Fig. 4). Although different matrices affected the detector response of palmitic acid-*d*_2_, no significant isotopic differences were found between solvent and DOM matrices (*P* = 0.8669 between solvent and SRFA and *P* = 0.4754 between solvent and marine DOM), and a sub-‰ SEM was achieved. These results indicate that the ion population, rather than the sample matrix, mostly affects the accuracy and precision of isotope analysis using UHRMS. The results also suggested that matrix effects and ionization processes did not induce isotope fractionation to the investigated intact fatty acids during ESI-UHRMS analysis of NOM or that any such fractionation was below the currently achievable sub-‰ accuracy with this method. Notably, in-source fragmentation may cause distinct isotope fractionations and requires particular caution (*18*). The bias from mass peak magnitude mismatch can be minimized by adjusting the sample concentration, infusion rate, or IAT.

Similarly, the δ^13^C values of octadecylphosphonic acid (ODPA) remained unaffected by matrix changes from terrestrial to marine, maintaining an SEM of 0.6‰, because of consistent averaged monoisotopolog mass peak magnitudes across samples (CV < 1%) (fig. S18). In conclusion, our results demonstrate that by targeting a consistent mass peak magnitude of the monoisotopologs, CSIA and MSIA can be reliably applied to molecules within complex mixtures like NOM. Nevertheless, CSIA or MSIA of the target analytes should be conducted using a single, consistent ionization mode during measurements, even when the compounds can be ionized under both positive and negative ESI conditions.

While the direct infusion analyses via a dual-inlet system expectedly resulted in a stable ion signal and optimal results, autosampler-based infusion inherently produced ion intensities that spanned approximately two orders of magnitude [noise (10^5^ A.U.) to stable signal (10^7^ A.U.)] (fig. S15). Given the (nonlinear) logarithmic relationship between measured δ^13^C values and mass peak magnitudes, less abundant peaks disproportionately influenced the δ^13^C mean (fig. S16). As a consequence, the SEM for replicates infused with an autosampler was larger than that of dual-inlet infused samples despite similar mass peak magnitudes and with double the number of carbon atoms (SEM of 0.8‰ under 2.8 × 10^7^ for palmitic acid with 16 carbon atoms and SEM of 0.6‰ under 2.6 × 10^7^ for caffeine monomer with 8 carbon atoms) (Fig. 4 and fig. S13). Nevertheless, autosampler infusion achieved sufficient accuracy and precision for ^13^C to reliably distinguish isotopic differences of several ‰. Similar issues related to transient signals apply to chromatographic peaks, and stabilizing the ion population using AGC, slowing down of the elution flow, or peak homogenization helps to reduce ion intensity fluctuations and related biases in IRs (*15*, *19*). A posteriori FTMS data processing, including data trimming (*2*, *13*), apodization, and absorption mode signal processing, may further improve precision.

### Calibration strategy for NOM molecules without reference materials

For molecules that lack comprehensive characterization and certified RMs, such as MFs in NOM, informative and robust MSIA results for the stable carbon isotope, expressed as Δδ^13^C values, can now be achieved using an intensity tuning strategy. However, the Δδ^13^C values alone limit the comparability of analytes across systems and laboratories, highlighting the need for a calibration strategy that anchors individual δ^13^C values to conventional scales such as VPDB. For organic molecules with available RMs, i.e., CSIA, the δ^13^C values with sub-‰ accuracy and precision can be achieved even for interlaboratory comparisons using at least one-point calibration (fig. S9). For CSIA, standard addition with ^13^C_1_-labeled standards can substantially reduce uncertainties arising from sample preparation and measurements. In contrast, for MSIA, standard addition with structurally identical RMs is not feasible because of the high structural diversity of NOM.

The success of the intensity tuning strategy offers a promising alternative for calibrating measured δ^13^C values using RMs that share the same number of carbon atoms as the target analyte. In theory, if the mass peak magnitudes of ^12^C*_n_* are identical, the corresponding intensity-dependent systematic errors in δ^13^C should also be consistent. Such universal RMs can be applied for one- or two-point calibration to standardize δ^13^C measurements across a range of MFs with the same carbon number, albeit with reduced accuracy compared to standard addition methods using structure-identical RMs. Nevertheless, a key challenge lies in understanding the potential influence of molecular mass on the observed δ^13^C.

The effects of the mass on the δ^13^C value measurements were evaluated by comparing oleic acid [molecular weight (MW), 282 Da; expected δ^13^C = −29.7‰] and ODPA (MW, 334 Da; expected δ^13^C = −30.9‰) that differed by 1.2‰ in δ^13^C. When analyzed under identical monoisotopolog peak magnitudes, both compounds yielded comparable δ^13^C values [3.6 ± 2.6‰ (SE) and 7.7 ± 3.5‰ (SE), *P* = 0.3299, Δδ^13^C = −4.1‰, 95% confidence interval of Δδ^13^C = −12.6 to 4.3‰] on the basis of 3600 spectra (fig. S17). These results indicate a maximum mass discrimination of 5.3‰ across a 50-Da mass range, roughly twice the uncertainty introduced by the inner precision (SE = 2.6‰). Therefore, δ^13^C values of analytes could be calibrated or at least approximated using RMs with known δ^13^C values and the same number of carbon atoms, even when their molecular masses differ (<50 Da). Assuming a linear dependence of isotope discrimination with mass difference, the resulting bias on the observed δ^13^C becomes negligible (<0.05‰) when the analyte and calibrant share the same nominal mass (<1 Da). Nonetheless, potential mass discrimination effects arising from molecular weight differences between analytes and calibrants require further investigation.

**Fig. 5.**
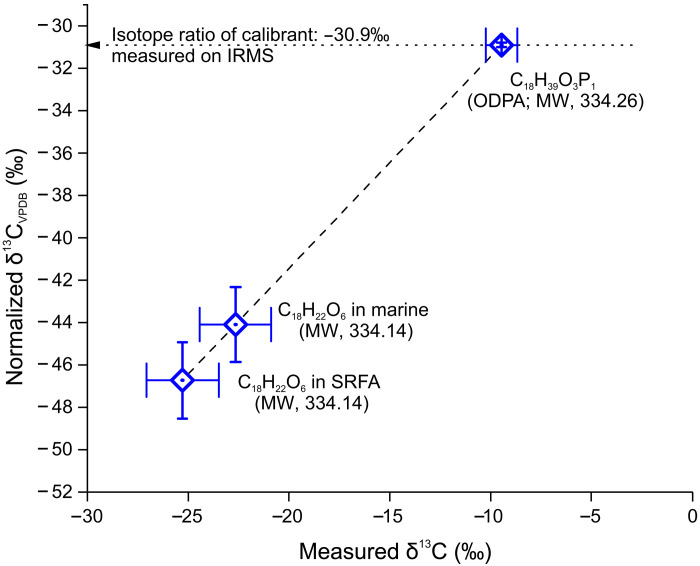
FT-ICR MS–derived δ^13^C for the MF C_18_H_22_O_6_ in NOM using one-point calibration. Performance of the one-point calibration realized with direct infusion (DI) via a dual-inlet system: ODPA (C_18_H_39_O_3_P_1_; δ^13^C_VPDB_ is expected at −30.9‰) was used as the calibrant and spiked into SRFA (3S101F) and the marine sample. The δ^13^C values of ODPA were predicted at −9.5 and −9.2‰ using linear regression models at the mass peak magnitude of 1 × 10^7^ (A.U.) (compare fig. S18B). The mass peak magnitudes of the MF C_18_H_22_O_6_ in both SRFA and the marine sample were tuned to a comparable level as ODPA (1.02 ± 0.03 × 10^7^ A.U., SD), with a CV < 3%. The error bars for other data points refer to the SE of δ^13^C values, except for the ODPA measured among both syringes for which the SEM was calculated. The short-dashed line indicates the 1:1 line.

On the basis of these findings, we obtained the isotopic composition of the DOM MF C_18_H_22_O_6_ using one-point calibration using ODPA (δ^13^C is expected at −30.9‰), which also contains 18 carbon atoms and has the same nominal mass (Fig. 5A). The δ^13^C values of C_18_H_22_O_6_ in different samples may be directly calibrated against an ODPA standard solution in ultrapure water given the identical mass peak magnitude of the monoisotopologs, after which the isotope variance across samples, i.e., Δδ^13^C, can be calculated and assessed. However, during method development, we observed that the marked matrix changes from ultrapure water to DOM destabilized the ionization of ODPA, leading to shifts in ODPA peak intensity and, hence, deviations from the initially matching intensity of the DOM MF C_18_H_22_O_6_ (similar to Fig. 3). As a more robust approach, we first spiked ODPA into both SRFA and marine DOM and measured them simultaneously with C_18_H_22_O_6_, with a mass difference of only 0.12 Da.

At the same averaged monoisotopolog intensity as C_18_H_22_O_6_ (10^7^), the δ^13^C value of ODPA was determined to be −9.4‰ (fig. S18). The δ^13^C values of C_18_H_22_O_6_ were measured at −25.3 ± 1.8‰ (SE) in SRFA and −22.7 ± 1.8‰ (SE) in the marine sample (*P* = 0.296, no statistical significance), yielding a Δδ^13^C at 2.7 ± 2.5‰ (SE) (Fig. 2A and fig. S19). Using ODPA for one-point calibration, the δ^13^C values of C_18_H_22_O_6_ could be calibrated to −46.8 ± 1.8‰ (SE) in SRFA and −44.2 ± 1.8‰ (SE) in the marine sample.

Unlike the marine marker MFs that exhibit more pronounced isotopic fractionations, C_18_H_22_O_6_ had the smallest Δδ^13^C value of 2.7‰ despite being determined with the highest inner precision (SE = 2.5‰). This suggests that the isotopic fractionation varies substantially among DOM MFs and samples, which calls for further instrumentational and method refinements for formulas with only small isotopic variances (<5‰). During this measurement, C_18_H_22_O_6_ exhibited averaged monoisotopolog intensities that were 2.3 and 7.2% higher in SRFA and the marine sample, respectively, compared to 10^7^ A.U. at which δ^13^C of OPDA was determined. A cautious correction was therefore applied to account for the intensity-dependent bias for measured δ^13^C values. On the basis of prior observations with the caffeine Na-dimer (4.0‰ δ^13^C deviation from a 14.1% difference in averaged intensity), the 2.3 and 4.2% higher intensities would be expected to introduce approximate −0.6 and −1.4‰ negative δ^13^C offsets to SRFA and the marine sample. Accounting for this adjustment, the measured δ^13^C values of C_18_H_22_O_6_ in SRFA and the marine sample could be further revised to −46.2 and −42.8‰, respectively, corresponding to a Δδ^13^C value of 3.4‰. Overall, this study demonstrates the feasibility of MSIA in complex mixtures like NOM, encompassing both (i) isotopic comparisons for the same MF across matrices (Δδ^13^C) and (ii) isotopic composition determinations via normalization or calibration against universal calibrants (δ^13^C_VPDB_) for interlaboratory comparison.

### Recommendation for CSIA and MSIA of molecules in NOM

Without intensity tuning, molecules across a broad mass range ionize competitively, resulting in substantial variability in the monoisotopolog mass peak magnitude. This variability leads to inaccurate δ^13^C values and also challenges, if not invalidates, calibration against RMs. However, this intensity-dependent variance can be strategically leveraged to obtain reliable and meaningful Δδ^13^C and even δ^13^C values.

1) If the averaged monoisotopolog mass peak intensity of a target analyte is matched among different samples (with CV < 2%), the comparison of δ^13^C values across samples is meaningful, and Δδ^13^C values can be obtained. This highlights the importance of intensity tuning as a strategy to mitigate concentration biases and matrix effects across different samples, thereby supporting the feasibility of CSIA and MSIA for organic molecules in complex mixtures such as NOM using UHRMS. Achieving comparable and consistent ^12^C*_n_* monoisotopolog intensities is one of the key aspects for accurate δ^13^C value measurements with UHRMS. This intensity tuning can be accomplished by adjusting the analyte concentration, flow rate of injection, AGC in Orbitrap instruments, or IAT in FT-ICR instruments. Careful inspection of transients (free induction decay) and mass spectra is crucial to detect ion population–related artifacts (space-charge effects and ion coalescence) or in-source fragmentation that can possibly bias the determined IRs (*8*, *18*). While direct infusion approaches (e.g., using a dual- or multi-inlet system) can still be considered a gold standard for ESI-UHRMS measurements of IRs, recent studies show prominent developments for using chromatographically separated compounds, leading to shorter transient signals (*2*, *19*). However, these approaches come with a higher experimental effort, increased measurement time, and data processing needs.

2) Rapid yet robust isotope analysis is achievable because of the inverse relationship between ion count and uncertainty: The uncertainty of the estimated δ^13^C value decreases by half with every fourfold increase in the mass peak magnitude (*8*). This reinforces the importance of maintaining high and consistent ion statistics for precise δ^13^C measurements. A narrow mass window is key to maximize and harmonize the mass peak magnitudes of targeted analytes across samples in the mass analyzer by continuous accumulation of selected ions (CASI) in FT-ICR or an advanced quadrupole selector in Orbitrap instruments. In conjunction with AGC, the quadrupole transmits a constant ion population within the small selected mass range into the Orbitrap, which partly alleviates the intensity variations of analytes from ionization efficiency changes over measurement time. Despite these improvements, broadband determination of IRs for intra- and intersample comparisons is still not possible.

3) Last, UHRMS-derived δ^13^C values can be anchored to the VPDB scale using universal RMs that share the same number of carbon atoms with the target DOM formula, provided that monoisotopolog peak intensities are matched through intensity tuning. This approach is supported by only small mass discrimination effects observed within a ±50-Da mass window, which remains well within the bounds of the inner measurement precision. Considering that NOM molecules typically contain 10 to 50 carbon atoms across a mass range of 1000 Da (*20*), ~40 RMs may be necessary to cover any 50-Da range (and 800 for the entire range of 1000 Da). Using such RMs, δ^13^C values could be assigned to multiple MFs within the same analytical run. In this regard, existing RMs can be further leveraged, some of which are developed with both depleted and enriched isotope abundances for two-point calibration (*21*, *22*). Additional RMs with distinct numbers of carbon atoms can also be synthesized on the basis of the principles outlined in this work. Fatty acid methyl ester RMs covering a range of carbon numbers are already accessible. These petrol-based materials are depleted in natural isotope abundance and could be used for one-point calibration. In the future, leveraging isotope labeled *m*/*z* (formula) identical RMs with close structural similarity to NOM compounds (*23*) could enable standard addition approaches and tests for structure-dependent matrix effects, thereby further improving measurement accuracy.

### Environmental implication

UHRMS has been successfully used to measure natural abundance stable carbon IRs, δ^13^C, of organic compounds with sub-‰ accuracy and precision. However, the susceptibility of CSIA to concentration biases and matrix effects has traditionally necessitated extensive prepurification, limiting its application to complex mixtures such as NOM, where sample heterogeneity and the limited accessibility of identical RMs pose additional challenges to δ^13^C determination at the MF level.

In this study, we demonstrated the feasibility of stable isotope comparison for MFs in complex organic matter. Considering the complex structural nature, we introduce the term MSIA to differentiate between true CSIA and the δ^13^C determined for molecular masses in complex NOM. We demonstrated that intensity-dependent variance primarily governs the inner precision of δ^13^C value measurements with UHRMS, which consequently also applies to other δ value measurements (e.g., δ^15^N, δ^34^S, and δ^18^O). It should be noted that for target formulas containing non-oxygen heteroatoms, higher mass resolving power is required to resolve corresponding isotopologs (e.g., M + 1: ^15^N; M + 2: ^34^S) from ^13^C isotopologs and other coexisting formulas with small mass differences. Ultimately, the highest resolving power of FT-ICR MS enables the clumped-isotope analysis, which has gained increasing prominence in (bio)geochemistry (*11*, *24*).

Following the intensity tuning strategy for monoisotopologs, MSIA enables a stable carbon isotope comparison of single MFs between samples, Δδ^13^C, and is reproducible even in the presence of concentration-dependent biases and matrix effects. This alleviates the need for stringent compound isolation and matrix matching protocols, which currently hindered CSIA for NOM molecules and opens possibilities of IR comparison analysis in complex mixtures. We determined Δδ^13^C values for four molecules in DOM across terrestrial and marine samples, revealing a broader spectrum of isotopic variance than recognizable from bulk δ^13^C values. Consistent with previous work, marker molecules that were found to be relatively more abundant in marine DOM consistently indicate ^13^C enrichment in the marine sample as compared to SRFA, while nonmarker formulas were isotopically indistinguishable. These results underscore the need to reassess source partitioning and mixing models on the basis of bulk δ^13^C values of DOC (*10*, *25*–*28*) and outline how MSIA may deliver crucial insights for geochemical research, e.g., to better constrain the amount of terrestrial organic matter in the ocean or the fate of permafrost carbon (*29*–*31*). Notably, as determined δ^13^C values for an MF in different NOM samples still represent a concentration-averaged value for a large number of isomers, a consistent shift in isomeric composition [and corresponding ionization efficiencies; (*32*, *33*)] across samples may lead to a possible bias in calculated Δδ^13^C values that requires further investigation, e.g., via polarity separation using LC-FT-ICR MS (*9*).

Sub-‰ precision in δ^13^C value measurement remains attainable through statistically robust sampling, enabled by high ion statistics. Sub-‰ accuracy is achievable with UHRMS through bracketing with chemically identical RMs. Here, we show that universal RMs that share the same number of carbon atoms as the target analyte (e.g., commercially available fatty acid methyl esters or formula-identical, synthetized compounds) offer the potential for calibrating NOM MFs. Notably, this study presents the determination of δ^13^C values for individual MFs in DOM, such as C_18_H_22_O_6_, with calibrated values of −46.8‰ in SRFA and −44.2‰ in marine surface water from the Weddell Sea. Extreme depletion in ^13^C of biomass or organic matter may be the result of excessive organic matter turnover (*34*–*36*), carbon reservoirs with limited atmospheric CO_2_ exchange (e.g., deep ocean) (*37*), or methanotrophically derived organic matter (*38*). Therefore, MF-specific δ^13^C values can pave the way forward in studying organic matter aging and processing across ecosystems, eventually contributing to the conundrum of long-term stable organic carbon pools on land and in the ocean. The universal standard IR measurement system presented here harnesses the full potential of UHRMS, extending its applicability beyond environmental sciences to diverse analytical fields including petroleomics, metabolomics, biogeochemistry, and forensics.

## MATERIALS AND METHODS

### Samples and chemicals

Different chemicals and samples with varying molecular mass and δ^13^C values were used (table S1). Except for caffeine (USGS63 and IAEA-600 RMs), bulk δ^13^C values of all samples and chemicals were determined using an EuroEA3000 element analyzer (HEKAtech, Germany) coupled to Thermo Fisher Scientific MAT 253 IRMS (Thermo Fisher Scientific, Germany), with uncertainties reported as SD. SRFA [SRFA III (3S101F)] was obtained from the International Humic Substances Society, and the powder was directly used for IRMS analysis. The marine sample was a methanolic solid-phase extract (Bond Elute PPL, Agilent) at 544 mg C/liter (see the “Sample description” section in the Supplementary Materials). For the IRMS analysis, the marine sample was dried with nitrogen gas. All samples were measured in triplicate, except for the marine sample, which was measured in duplicate. For the measurements of δ^13^C values with FT-ICR MS, all samples were dissolved in ultrapure water and methanol (50:50, v:v) to enhance ionization efficiency.

### Dual-inlet system for direct-infusion FT-ICR MS

Using a dual-inlet system (*11*), two types of analytes were alternately introduced into the mass spectrometer using a syringe pump (Fusion 4000X, Chemyx, Stafford, TA) and a six-port/two-position divert valve (Rheodyne, IDEX Health & Science LLC, Northbrook, IL). Samples from two syringes (1002 TLL, Hamilton) were pumped simultaneously at a constant flow rate (240 μl/hour). By switching the valve, the injection of sample alternated by syringe selection every 20 min (sample 1/sample 2/sample 1/...) over a total duration of 600 min, unless otherwise specified.

### Autosampler-aided direct infusion

To simplify the test for matrix effects using three samples, an autosampler with a 100-μl sample loop was used with an ultrahigh-pressure binary pump of an ultrahigh-performance liquid chromatography system (Elute, Bruker Daltonics, Billerica, MA). The mobile phase consisted of ultrapure water (A: 50%) and methanol (B: 50%) delivered at 10 μl/min, with 11.9 min of data recorded per injection. Source settings matched those used for the dual-inlet infusion. Sample batches were run in an alternating order to mimic sample/standard bracketing with three replicates per sample (sample 1/sample 2/sample 3/...).

### Description of FT-ICR MS datasets

Eight datasets were acquired by FT-ICR MS (7 T scimaX 2xR, Bruker Daltonics Inc., Billerica, MA) operated in serial mode. Unless otherwise specified, samples were infused via the dual-inlet system. Data were acquired in negative and positive ion modes with an ESI source (Apollo II, Bruker Daltonics; capillary voltage: 4.5 kV for positive mode and 4.0 kV for negative mode) in full profile magnitude mode with a transient size of 4 MWord (∼1.6-s free induction decay). The IAT varied from 20 to 600 ms. The detection mass range was set to full mass range with selected *Q* isolation windows using the CASI mode (table S2). For this, each isolation window was set to 5 Da and centered between the monoisotopic ^12^C*_n_* peak and the ^12^C_*n*−1_^13^C_1_ peak of the targeted MFs. The mass resolving power (*m*/Δ*m*, full width at half-maximum) at *m*/*z* 400 was ~500,000 and sufficient to resolve all major NOM ions in the considered mass range. Four scans were averaged into one mass spectrum, except for the Caffeine_Int_differ dataset (see below), which required single scan acquisition. Lock mass calibration was performed to the averaged spectra using masses of the ^12^C*_n_* monoisotopolog and ^13^C_1_ isotopolog of analytes.

1) Caffeine_Int_differ: Caffeine standards (USGS63 and IAEA-600) were dissolved in ultrapure water and loaded at an identical concentration of 0.5 mg/liter. Measurements were acquired for the full mass range (147 to 1000 Da) using ESI+. Sample injections alternated every 10 min.

2) Caffeine_Int_match: Caffeine standards (USGS63 and IAEA-600) in ultrapure water were loaded at an identical concentration of 0.5 mg/liter. Measurements were done in multi-CASI mode (with multiple mass centers at *m*/*z* 217.57 and 411.65) under ESI+, with intensities tuned to be comparable [(1.74 ± 0.02) × 10^7^ (A.U.) and the CV was 1.1% for the Na-monomer [M + Na]^+^; (8.14 ± 0.6) × 10^6^ (A.U.) and 7.1% for the Na-dimer [2 M + Na]^+^].

3) Oleic_acid_Int_match: An oleic acid sample dissolved in ultrapure water with a concentration of 0.013 ng/liter was split and loaded into the two syringes, and the mass peak magnitude increased over time because of changes in instrumental sensitivity over 10-hour measurement time. Measurements were acquired in CASI mode centered at *m*/*z* 282.76 using negative ESI mode (ESI−).

4) Caffeine_STD_matrix: The caffeine standard USGS63 was dissolved in an aqueous solution of SRFA (5 mg C/liter) to achieve a concentration of 0.5 mg/liter. Measurements were conducted in multi-CASI mode (*m*/*z* 195.59, 217.57, and 411.65) under ESI+.

5) SRFA_Marine_marker: A DOM sample with terrestrial origin, i.e., SRFA, and a marine DOM extract obtained from Weddell Sea surface waters with negligible terrestrial inputs were used (compare the “Sample description” section in the Supplementary Materials). Three target MFs present in both samples were selected on the basis of their high positive correlation between their relative mass peak abundances and the bulk δ^13^C of DOC (*39*). We refer to those MFs as a “marine marker.” In our work, three marine markers from the set of 40 (C_19_H_22_O_10_, C_20_H_26_O_9_, and C_20_H_24_O_9_) were selected for their high abundance in our sample spectra and moderate molecular weights (410.1213, 410.1577, and 408.1420 Da, respectively) that match the commonly reported average molecular weight of recalcitrant DOM molecules (*2*). SRFA was prepared at concentrations of 5 mg C/liter. The marine sample was diluted to concentrations of 1 mg C/liter for C_19_H_22_O_10_ and at concentrations of 2 mg C/liter for C_20_H_26_O_9_ and C_20_H_24_O_9_ to harmonize mass peak intensities of the three marine markers. Measurements were done in CASI mode under ESI−. The dataset consists of three measurements obtained individually for the three markers (table S2). The isolation windows were centered at 409.61, 409.65, and 407.63 Da for C_19_H_22_O_10_, C_20_H_26_O_9_, C_20_H_24_O_9_, respectively.

6) C18_mass_differ: Oleic acid (C_18_H_34_O_2_; MW = 282 Da) and ODPA (C_18_H_39_O_3_P_1_; MW = 334 Da), both with comparable δ^13^C values (table S1), were prepared at 0.13 ng/liter in ultrapure water. Measurements were done with ESI− in multi-CASI mode (table S2) and isolation windows centered at 281.75 and 333.76 Da for C_18_H_34_O_2_ and ODPA, respectively.

7) NOM_calibration: SRFA and the marine sample were loaded at concentrations of 5 and 10 mg C/liter. An MF C_18_H_22_O_6_ was measured together with ODPA. C_18_H_22_O_6_ was selected because of its apparent recalcitrance in oceanic environments indicated by a positive correlation of its relative mass peak abundance with the bulk Δ^14^C of DOC (suggesting relative accumulation with DOM age) and its lack of a significant correlation with the bulk δ^13^C of DOC (*39*, *40*). ODPA, serving as a calibrant, was spiked into both samples to a final concentration of 0.013 ng/liter to match the averaged mass peak intensity of the MF C_18_H_22_O_6_. The samples were infused with a dual-inlet system, and the measurements were done with an IAT of 400 ms in multi-CASI mode under ESI− with isolation windows centered at 333.63 Da for C_18_H_22_O_6_ and 333.76 Da for ODPA.

8) Palmitic_acid_matrices_LC: Palmitic acid-*d*_2_ was prepared in different matrices at varying concentrations to achieve comparable monoisotopolog mass peak magnitudes during FT-ICR MS measurements. Palmitic acid was prepared at 0.06 mg/liter in pure solvent (MeOH/MQW; 50:50 v/v), at 0.1 mg/liter in the presence of SRFA (5 mg C/liter, in MeOH/MQW; 50:50 v/v), and at 0.15 mg/liter in the presence of marine DOM (10.9 mg C/liter, in MeOH/MQW; 50:50 v/v). Measurements were done in CASI mode under ESI−, with isolation windows centered at 257.75 Da. All samples were injected via an autosampler.

Further details for all datasets are described in the Supplementary Text (“Sample description” section and table S2).

### Data processing

Unprocessed line spectrum data in ASCII format were exported from DataAnalysis 6.0 (Bruker Daltonics). A custom R script was developed for the extraction of ion intensity from ASCII files and for further processing. The R script (IsoTrack, version 0.1.0) and full data are accessible from https://doi.org/10.5281/zenodo.18999537 and https://doi.org/10.48758/ufz.16017.

### Calculation of δ^13^C values with ^12^C*_n_* monoisotopolog and ^12^C_*n*−1_^13^C_1_ isotopolog peaks

The IR and δ^13^C values were calculated on the basis of raw peak intensities of the ^12^C*_n_* monoisotopolog and ^12^C_*n*−1_^13^C_1_ isotopolog pair for an organic molecule with carbon number of *n* and are reported relative to the VPDB scale according to Eq. 1. Detailed proofs are provided in the Supplementary Text (“Calculation of δ^13^C from peak intensities in UHR mass spectra” section) (*2*, *21*).

δ13C=(Intesity13C1=(n1)×p1×(1−p)n−1Intensity12Cn=(nn)×p0×(1−p)nn×IRStandard−1)×1000‰=(IRSample=p(1−p)IRStandard−1)×1000‰ (1)

where *n* represents the total carbon number in the molecules, *p* is the proportion of ^13^C, and (1 − *p*) corresponds to the proportion of ^12^C. The same equation (Eq. 1) as in previous studies was applied (*2*, *6*, *12*), considering only the ^13^C (proportion = 0.011057, *p*) and ^12^C (proportion = 0.988943, 1 − *p*) isotopes in VPDB [*R*(^13^C/^12^C)_VPDB_ = 0.0111802], as the trace natural abundance of ^14^C (1.2 × 10^−12^) is negligible in the recent organic carbon pools (*41*, *42*). Excluding ^14^C (equivalent to a sample/compound Δ^14^C value of −1000 ‰) results in a deviation of only 12.2 parts per billion (1.22 × 10^−8^) in δ^13^C, far below the parts per million–level precision achievable with any commercial IRMS (fig. S1).

The IR can also be obtained by summing all ^12^C and ^13^C atoms from the monoisotopolog and all isotopologs mass peaks (Eq. 2), yielding a mathematically equivalent IR to that obtained using only the ^13^C_1_-^12^C*_n_* isotopolog pair (Eq. 1). Detailed proofs are provided in the Supplementary Text (“Calculation of δ^13^C from peak intensities in UHR mass spectra” section).

δ13C=(IRSample=Sum of intensity13C in all isotopologsSum of intensity12C in all isotopologsIRStandard−1)×1000‰=(IRSample=∑0nmn×(nm)×(1−p)n−m×pm∑0nn−mn×(nm)×(1−p)n−m×pmIRStandard−1)×1000‰=(IRSample=p(1−p)IRStandard−1) ×1000‰ (2)

where *n* represents the total number of carbon atoms in the MF, and *m* represents the number of ^13^C atoms in the multiply-substituted isotopologs. It can be shown that Eqs. 1 and 2 are mathematically equivalent. In practice, however, δ^13^C values in previous studies (*2*, *6*, *12*), as well as in this work, are calculated using Eq. 1 based only on the most abundant ^13^C_1_ and ^12^C*_n_* peaks. This choice is made for analytical reasons, because multiply-substituted isotopologs containing more than four ^13^C atoms are typically below the signal-to-noise threshold for peak detection and are therefore not observable in UHR mass spectra (their peak intensities are <1% relative to the ^12^C*_n_* peak for molecules containing fewer than 50 carbon atoms). Consequently, the use of Eq. 1 does not introduce additional systematic biases. In contrast, applying Eq. 2 without accounting for undetected multiply-substituted isotopologs would inevitably introduce carbon number–dependent systematic errors.

Caution should be given when selecting equations to derive theoretical isotope abundances for elements other than carbon, particularly for sulfur isotopes (^33^S and ^36^S) or in systems containing multiple major stable isotopes (e.g., clumped isotopologs). All data points were visualized on a base 10 logarithmic scale as δ^13^C follows a logarithmic distribution arising from the ratio scale of absolute ion counts of the ^12^C*_n_* monoisotopolog and ^12^C_*n*−1_^13^C_1_ isotopolog (see the “Observation of isotope ratio on a logarithmic scale” section in the Supplementary Text) (*43*).

### Analysis of error

Performing CSIA on molecules and MFs within NOM presents substantial challenges and requires a comprehensive understanding of potential error sources. First, internal errors were considered: Each individual analysis of a molecule spans up to 600 min, during which ~2500 spectra per sample or standard were recorded. The SD and SE of δ^13^C values across these consecutive spectra were calculated to assess the internal error and, hence, the internal precision. Second, the intermediate precision was examined, which refers to the precision across multiple analyses of the same sample over different runs. The intermediate precision was quantified as the SEM of the mean δ^13^C values obtained from several measurements. For instance, when the averaged mass peak magnitude of the monoisotopolog reached comparable values at 10^7^ A.U., the cumulative means of δ^13^C were −4.5 ± 5.6‰ (SE, *n* = 287) and −3.7 ± 4.9‰ (SE, *n* = 324) for oleic acid (Fig. 3D), and the corresponding SEM between the two means was 0.4‰.

### Nomenclature

Isotope variance of analytes across samples was assessed as differences of measured δ values (Δδ), e.g., Δδ^13^C for stable carbon isotopes. While the analytical workflow for isotope analysis is identical for single compounds (CSIA) and the MFs in NOM (MSIA), the δ^13^C values for exact masses in NOM reflect the concentration-averaged δ^13^C ratios of all structural isomers and therefore may not be directly comparable with CSIA results. For better clarity, we use CSIA only for distinct compounds and RMs and MSIA when molecules in NOM are involved.
