# Supplementary material for: Determination of stable carbon isotope ratios for molecules in natural organic matter using ESI FT-ICR MS
Source: Sci Adv. 2026 Jun 17;12(25):eaee5238. doi: 10.1126/sciadv.aee5238 (PMC13274584; doi:10.1126/sciadv.aee5238)
Supplement: Supplementary file 1 — Supplementary Text Figs. S1 to S19 Tables S1 to S3 [file sciadv.aee5238_sm.pdf]

Science Advances
AAAS

Supplementary Materials for

Determination of stable carbon isotope ratios for molecules in natural organic matter using ESI FT-ICR MS

Shuxian Gao *et al.*

Corresponding author: Shuxian Gao, shuxian.gao@ufz.de; Oliver J. Lechtenfeld, oliver.lechtenfeld@ufz.de

Sci. Adv. **12**, eaee5238 (2026)
DOI: 10.1126/sciadv.aee5238

This PDF file includes:

Supplementary Text
Figs. S1 to S19
Tables S1 to S3

Supplementary Text

Calculation of $\delta^{13}C$ from peak intensities in UHR mass spectra.

To calculate the expected relative abundance of $^{13}C_1$ isotopologue peaks, accurate carbon isotope relative abundance (percent) values obtained from IRMS were multiplied by the total number of carbon atoms of interest in a molecule. This is a special case of the binomial probability distribution which is used to estimate the abundance of isotopologues (e.g., A+1 peak, A+2 peak) in a molecule with a total of m atoms of ^{13}C (e.g., $^{13}C_1$ peak, $^{13}C_2$ peak) (*12*):

$$P(m) = \binom{n}{m} p^m (1-p)^{n-m} = \frac{n!}{m!(n-m)!} p^m (1-p)^{n-m} \qquad \text{(eq. 1)}$$

where ***m*** is the isotopic mass (isotopologue) peak (*m* = 0 being the monoisotopic peak A and ***m*** = 1 is the A+1 peak, etc.), ***n*** is the total number of carbon atoms, and $0 < \boldsymbol{p} < 1$ is the proportion of the ^{13}C which refers to accepted stable carbon isotope values (derived from the reference material VPDB with $p = 0.0110566$) (*41*) in our manuscript. Using these abundances, the VPDB referenced bulk isotope ratio is calculated as $IR(^{13}C/^{12}C) = 0.0111802$.

The theoretical (expected) relative abundance (RA) of each isotopologue peak (m>0) to $^{12}C_n$ (m=0) can be described using the binomial probability distribution as:

$$Expected\ RA(m) = \frac{P(m)}{P(0)} \qquad \text{(eq. 2)}$$

The previous study (*6*), and in our manuscript, the most abundant $^{13}C_1$ isotopologue peak was used, and the RA can be expected as:

$$Expected\ RA(1) = \frac{P(1)}{P(0)} \qquad \text{(eq. 3)}$$

with P(1) and P(0) described as:

$$P(1) = \frac{n!}{1!(n-1)!} p^1 (1-p)^{n-1} = np(1-p)^{n-1} \quad \text{(eq. 4)}$$

$$P(0) = \frac{n!}{0!(n-0)!} p^0 (1-p)^{n-0} = (1-p)^n \qquad \text{(eq. 5)}$$

Insert eq. 4 and eq. 5 into eq. 3 and we have:

$$Expected\ RA(1) = \frac{P(1)}{P(0)} = \frac{np(1-p)^{n-1}}{(1-p)^n} = n\frac{p}{(1-p)} \qquad \text{(eq. 6)}$$

Hence we can retrieve the isotope ratio IR from RA(1):

$$IR = \frac{RA(1)}{n} = \frac{p}{(1-p)} \qquad \text{(eq. 7)}$$

Therefore, the $\delta^{13}C$ value can be calculated as:

$$\delta^{13}C = \left(\frac{IR_{sample}}{IR_{standard}} - 1\right) \times 1000\ (‰) \qquad \text{(eq. 8)}$$

Here $IR_{sample} = \frac{\frac{Intensity\ ^{13}C_1}{Intensity\ ^{12}C}}{n}$, and $IR_{standard} = \frac{p}{(1-p)} = IR\left(\frac{^{13}C}{^{12}C}\right) = 0.0111802.$

$$\delta^{13}C = \left(\frac{\frac{Intensity\ ^{13}C_1}{Intensity\ ^{12}C}}{n\frac{p}{(1-p)}} - 1\right) \times 1000\ (‰) \qquad \text{(eq. 9)}$$

$$\delta^{13}C = \left(\frac{IR_{sample}}{\frac{n\times p}{(1-p)}} - 1\right) \times 1000‰ = \left(\frac{IR_{sample}}{n \times IR_{standard}} - 1\right) \times 1000‰ \qquad \text{(eq. 10)}$$

Impact of ^{14}C on calculated $\delta^{13}C$ values.

It should be noted that in the calculation of $\delta^{13}C$, only ^{13}C and ^{12}C isotopes are considered, with the proportion of ^{12}C expressed as (**1-*p***) in eq. 1 and ^{14}C is omitted. To evaluate the potential influence of ^{14}C, we also assessed its contributions at natural abundance (1.2×10^{-12}) to the expected relative isotope abundance of the $^{13}C_1$ isotopologue peak RA(1). In this assessment, the proportions of ^{13}C, ^{14}C and ^{12}C are **p** (0.0110566), **q** (1.2×10^{-12}), **j** (0.9889434), and p+q+j = 1.
Eq. 5 is now written as:

$$P(0)' = \frac{n!}{0!(n-0)!}p^0(j)^{n-0} = (j)^n \qquad \text{(eq. 5')}$$

Hence the RA(1) from eq. 6 is now expressed as:

$$ExpectedRA(1)' = \frac{P(1)}{P(0)} = \frac{np(1-p)^{n-1}}{(j)^n} \qquad \text{(eq. 6')}$$

The comparison of theoretical isotopologue abundances derived from **eq. 6** (excluding ^{14}C) and **eq. 6'** (including ^{14}C) is shown in Fig S1. The omission of ^{14}C results in a deviation of only 12.2 ppb (1.22×10^{-8}) in the relative abundance of isotopologue peaks for molecules containing 30 carbon atoms - far below the ppm-level precision achievable with any commercial IRMS. Hence, exclusion of ^{14}C in $\delta^{13}C$ calculations using UHRMS is scientifically justified.

Calculation of $\delta^{13}C$ values from all isotopologue species.

The isotope ratio can also be retrieved by summing all ^{12}C and ^{13}C atoms from all the monoisotopologue and isotopologues mass peaks, which yields an isotope ratio mathematically equivalent to the results using only isotopologue pairs of $^{13}C_1$ and $^{12}C_n$. In our study, only $^{13}C_1$ and $^{12}C_n$ are used to calculate the isotope ratios and without no extra systematic biases. The equivalency is proofed below.

According to probabilistic distribution, the multiply-substituted $^{13}C_m$ isotopologues in an organic molecule with ***n*** carbon atoms can be written as:

$$P(m) = \binom{n}{m}p^m(1-p)^{n-m} = \frac{n!}{m!(n-m)!}p^m(1-p)^{n-m} \qquad \text{(eq. 1)}$$

in which we have both ^{12}C and ^{13}C atoms. To calculate the sum of ^{12}C and ^{13}C atoms, we can separate the contribution according to both isotopes from these isotopologues as:

$$RA(m, 13C) = \frac{m}{n} \times \binom{n}{m} \times (1-p)^{n-m} \times p^m \qquad \text{(eq. 11)}$$

$$RA(m, 12C) = \frac{n-m}{n} \times \binom{n}{m} \times (1-p)^{n-m} \times p^m \qquad \text{(eq. 12)}$$

To sum up, the ^{13}C and ^{12}C atoms form all the isotopologues are expressed as:

$$\sum_0^n RA(m, 13C) = \sum_0^n \frac{m}{n} \times \binom{n}{m} \times (1-p)^{n-m} \times p^m \qquad \text{(eq. 13)}$$

$$\sum_0^n RA(m, 12C) = \sum_0^n \frac{n-m}{n} \times \binom{n}{m} \times (1-p)^{n-m} \times p^m \qquad \text{(eq. 14)}$$

Hence the IR(^{13}C/^{12}C) is calculated as:

$$IR\left(\frac{13C}{12C}\right) = \frac{\sum_0^n RA(m,13C)}{\sum_0^n RA(m,12C)}$$

$$IR\left(\frac{13C}{12C}\right) = \frac{\frac{0}{n}\times\binom{n}{0}\times(1-p)^{n-0}\times p^0 + \sum_{m=1}^{n}\frac{m}{n}\times\binom{n}{m}\times(1-p)^{n-m}\times p^m}{\sum_{m=0}^{n-1}\frac{n-m}{n}\times\binom{n}{m}\times(1-p)^{n-m}\times p^m + \frac{n-n}{n}\times\binom{n}{n}\times(1-p)^0\times p^n}$$

$$IR\left(\frac{13C}{12C}\right) = \frac{\sum_{m=1}^{n}\frac{m}{n}\times\frac{n!}{m!\times(n-m)!}\times(1-p)^{n-m}\times p^m}{\sum_{m=0}^{n-1}\frac{n-m}{n}\times\frac{n!}{m!\times(n-m)!}\times(1-p)^{n-m}\times p^m}$$

$$= \frac{\sum_{m=1}^{n}\frac{(n-1)!}{(m-1)!\times(n-m)!}\times(1-p)^{n-m}\times p^m}{\sum_{m=0}^{n-1}\frac{(n-1)!}{m!\times(n-m-1)!}\times(1-p)^{n-m}\times p^m}$$

$$= \frac{\sum_{m=1}^{n}\binom{n-1}{m-1}\times(1-p)^{(n-1)-(m-1)}\times p^{m-1}\times p}{\sum_{m=0}^{n-1}\binom{n-1}{m}\times(1-p)^{(n-1)-m}\times p^m\times(1-p)}$$

$$= \frac{\sum_{m=1}^{n}\binom{n-1}{m-1}\times(1-p)^{(n-1)-(m-1)}\times p^{m-1}}{\sum_{m=0}^{n-1}\binom{n-1}{m}\times(1-p)^{(n-1)-m}\times p^m} \times \frac{p}{1-p} \qquad \text{(eq. 15)}$$

Substitute k = m-1, then m = 1 means k = 0, and m=n means k = n-1, hence eq. 15 can be expressed as:

$$= \frac{\sum_{k=0}^{n-1}\binom{n-1}{k}\times(1-p)^{(n-1)-k}\times p^k}{\sum_{m=0}^{n-1}\binom{n-1}{m}\times(1-p)^{(n-1)-m}\times p^m} \times \frac{p}{1-p}$$

$$= \frac{(1-p+p)^{n-1}}{(1-p+p)^{n-1}} \times \frac{p}{1-p}$$

$$= \frac{p}{1-p} \quad \text{(eq. 16)}$$

And δ^{13}C is still expressed the same by eq.10:

$$\delta^{13}C = \left(\frac{IR_{sample}}{IR_{standard}} - 1\right) \times 1000\ (‰) \qquad \text{(eq. 10)}$$

While the isotope ratios can be mathematically retrieved by summing all ^{13}C and ^{12}C atoms from all the isotopic fine structures, the multiply-substituted ^{13}C isotopologues won't be always visible in the UHR mass spectra due to low expected peak intensities below the peak detection

limit S/N. In contract, the omission loss of undetected isotopologues in the isotope analysis by using the sum of all ^{13}C and ^{12}C atoms (eq. 16) will inevitably introduce biases.

Due to low peak intensities for molecules containing more than one ^{13}C, we focused on the isotope pairs with the largest intensities which are generally molecules containing only ^{12}C (monoisotopologue) and one ^{13}C ($^{13}C_1$ isotopologue) for NOM molecules of molecular weight (<1000 Da).

Of note, the presence of multiple stable isotopes (e.g., ^{33}S and ^{36}S in $\delta^{34}S$) or clumped-isotope calculations using multiple stable isotopes necessitates careful selection of equations used to derive theoretical isotopologue abundances.

Observation of isotope ratio on a logarithmic scale.

ICR and Orbitrap mass detectors respond to time-domain excitation and produce analogue signals in the time-domain (image current). As a result, the raw intensity output in FTMS does not precisely reflect the actual concentration of the analyte in the sample (*12*, *14*).

However, FTMS analyzers exhibit a linear response to the magnitude of excitation, which is proportional to the ion population within the mass analyzer. This property allows us to evaluate the accuracy of the isotope ratio measurements by examining the relationship between intensities of the monoisotopologue and its corresponding isotopologue (eq. 11):

$$IR_{FTMS} = \frac{I_{M+1}}{n \times I_M} \quad \text{(eq. 11)}$$

Where I_{M+1} and I_M refer to the intensities of the $^{12}C_n$ monoisotopologue and $^{12}C_{n-1}{}^{13}C_1$ isotopologue in FTMS, and ***n*** refers to the carbon number of the molecules. If we apply a logarithm to the base of 10, we obtain a linear relationship (eq. 12).

$$Log_{10}(I_{M+1}) = Log_{10}(I_M) + Log_{10}(n) + \boldsymbol{Log_{10}(IR_{FTMS})} \quad \text{(eq. 12a)}$$

$$Log_{10}(I_{M+1}) - Log_{10}(n) = Log_{10}(I_M) + \boldsymbol{Log_{10}(IR_{FTMS})} \quad \text{(eq. 12b)}$$

By performing a linear regression where

$$Y = Log_{10}(I_{M+1}) \text{ and } X = Log_{10}(I_M) + Log_{10}(n), \quad \text{(eq. 13a)}$$

or alternative,

$$Y = Log_{10}(I_{M+1}) - Log_{10}(n) \text{ and } X = Log_{10}(I_M), \quad \text{(eq. 13b)}$$

the model ideally yields a slope of 1. The intercept represents the isotope ratio and can be used to calculate the $\delta^{13}C$ similar to eq. 10.

It should be noted that the isotope ratio could also be derived from a linear regression using raw intensity values on the original absolute scale (i.e., I_{M+1} and I_M per carbon). In such case, the expected linear regression model is:

$$I_{M+1 \text{ per C}} = IR_{FTMS} \times I_M, \quad \text{(eq. 14)}$$

Here, the slope represents IR_{FTMS}, and the intercept is 0. However, due to the small magnitude of IR_{FTMS} (e.g., 0.0111802 for VPDB), distinguishing significant differences between analytes becomes challenging. For a better observation of the isotope ratios and their precision and accuracy on FTMS, this study visualizes the data on logarithmic (base 10) scale.

It should be noted that there might be potential errors-in-variables (EIV) biases in the regression $Y = \log(I_{m+1}) - \log(n) = \log(I_m) + A$. In this regard, model II regression is recommended.

Nevertheless, in our study, this linear form and its logarithmic representation were used solely to **visualize** the proportional relationship between the measured isotope peak intensities and the number of carbon atoms (n), rather than to obtain quantitative parameter estimates. All $\delta^{13}C$ values were calculated with eq. 10 based on the raw intensity scale. No regression coefficients, i.e. isotope ratio A in the equation 13, derived from this equation were used in subsequent analyses or interpretations. Therefore, the possible EIV bias does not affect any of the reported results or conclusions.

Sample description

Samples in this study were analyzed under different FT-ICR instrumental tuning conditions, using direct infusion (DI) in serial mode and a 6-port 2-position valve switching approach to alternate between two solutions. The initial flow rate was set to 4 μL/min, corresponding to a total volume of 2.4 mL over 600 mins, but this is changeable for intensity tuning needs. Each dataset comprised spectra averaged over four scans, except the *Caffeine_int_differ* dataset for which single-scan spectra were recorded. For molecular formula specific isotope analysis (MSIA), two distinct NOM samples were analyzed: SRFA, representing terrestrial NOM and a marine sample with negligible terrestrial inputs. The marine sample (PS-129-67-1) was collected the Weddell Sea (68.86519° S, 17.94656° W; 11 m depth; sampled on 04 April 2022), and 500 ml NOM was extracted and concentrated from a 950 L sea water (DOC of 46 μM) using 17g Bond Elute PPL solid phase extraction (SPE) cartridge (Agilent) operated by a PTFE pump at a flow rate of 333 mL/min, yielding a SPE-DOC concentration in the final methanolic extract of 544 mg-C/L and extraction efficiency of ~50%. The samples and data acquisition are described in Table S1 and Table S2.

Caffeine_Int_differ dataset: In this sample, two caffeine standards (i.e., USGS63 and IAEA-600) were dissolved into ultrapure water and introduced into the two syringe channels. Contaminants, primarily fatty acids were inevitably introduced and detected (raw mass spectrum is shown below; Fig. S2). Their randomness, variable intensity levels, and differing carbon numbers were useful for the assessing spectral accuracy in a near matrix free environment. The molecular formulas (MFs) are listed below in Table S3.

Caffeine_Int_match dataset: Caffeine standards (USGS63 and IAEA-600) measured in ultrapure water, introduced through two syringes, and tuned for comparable intensities

Oleic_acid_Int_match dataset: The applicability of intensity tuning to account for the concentration bias was further assessed. The same sample of oleic acid was split into two syringes, and the amounts of ionized analytes was tuned via changing injection flow rates, corresponding to different concentrations (Figure 2A-B). As the intensity of the monoisotopologue continuously increased, the cumulative $\delta^{13}C$ values continued to decrease and failed to converge with time (Figure 2C). However, when the cumulative means of $\delta^{13}C$ values were calculated sequentially by increasing monoisotopologue intensity (rather than by time), they showed remarkable consistency (Figure 2D). The differences in measured $\delta^{13}C$ values between syringes at the same intensity level were predicted to range from -2.9‰ to 4.6‰, which falls within the uncertainty range at the given intensity level (Fig. S10).

Caffeine_STD_Matrix dataset: The reproducibility of the intensity tuning strategy for caffeine in different matrices were first assessed in ultrapure water and 5 mg-C/L SRFA using ESI+. In ultrapure water, the ionization of the Na-monomer and Na-dimer was rather stable with a relative error of 1% in averaged monoisotopologue peak intensity (Fig. S12A & Fig. S13). Consequently, the measured $\delta^{13}C$ values were highly reproducible, with deviations of 1.01‰ and

1.15‰ for the Na-monomer and Na-dimer, respectively. In SRFA, caffeine was ionized as a Na-monomer, Na-dimer and H-monomer due to the matrix, and only sodium adducts were analyzed (Fig. S12B & Fig. S14). While the relative errors were larger, the repeatability was 2.35‰ and 1.38‰ difference in $\delta^{13}C$ values for Na-monomer and Na-dimer with relative errors of 2.62% and 4.74% for the intensity of the monoisotopologues, respectively. The Na-dimer reproduced better, despite a larger relative error, because the variance in intensity was mitigated by its doubled carbon number compared to the monomer. In conclusion, the measured $\delta^{13}C$ values were reproducible when the intensities of monoisotopologue were matched.

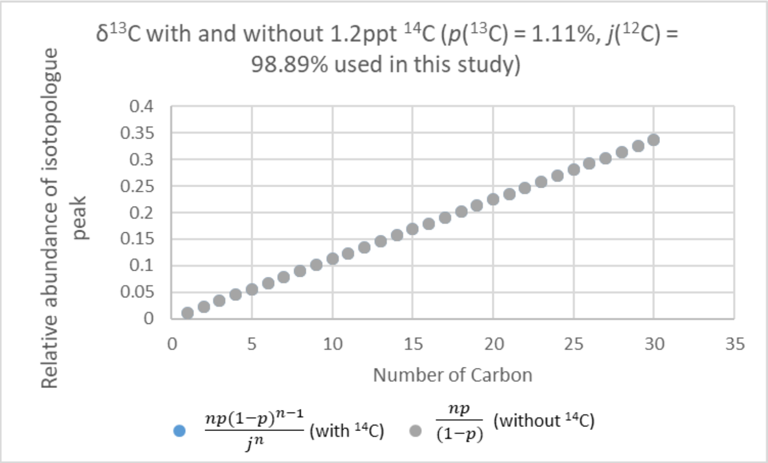

Fig. S1. Theoretical relative isotope abundance of $^{13}C_1$ isotopologue peak versus number of C atoms. Color code: considering the natural ^{14}C abundance of 1.2 ppt, blue, in comparison to equation without ^{14}C abundance, gray. The exclusion of ^{14}C leads to only 12.2 ppb deviation (1.22×10^{-8}) in the relative abundance of isotopologue peaks for the molecules with 30 carbon atoms - far below the ppm-level precision achievable with any commercial IRMS. Blue dots hidden behind grey dots.

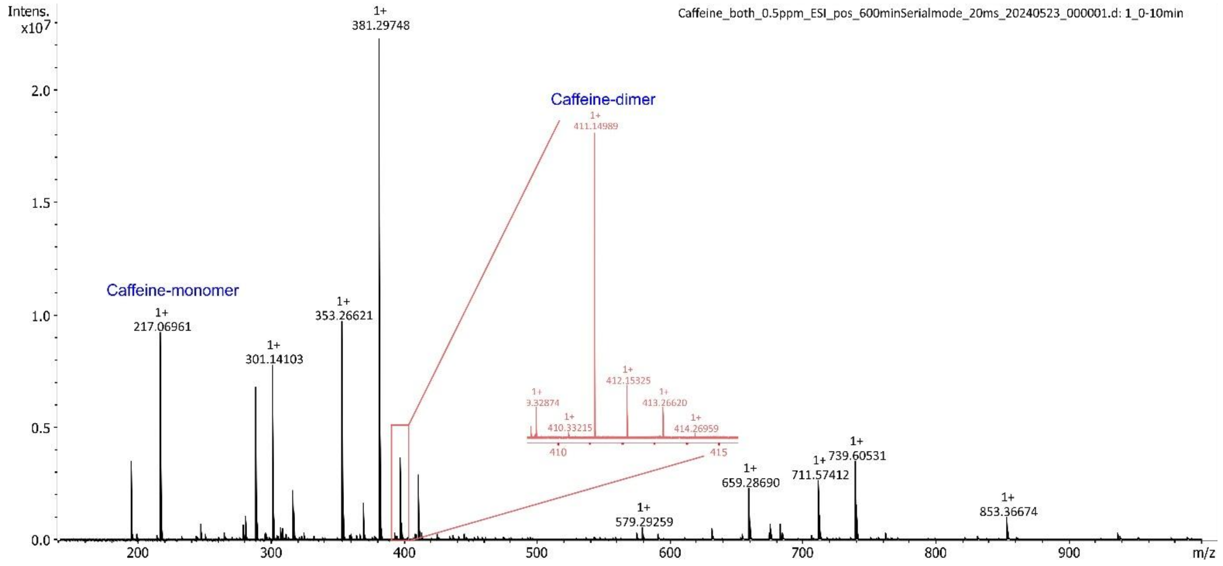

Fig. S2. FT-ICR mass spectrum of the *Caffeine_Int_differ* sample. The caffeine monomer was detected at m/z 217 as a sodium adduct, and the caffeine dimer was detected at m/z 411 as a sodium dimer. The compound spectrum was averaged from spectra between 0-10 minutes, equivalent to 375 scans.

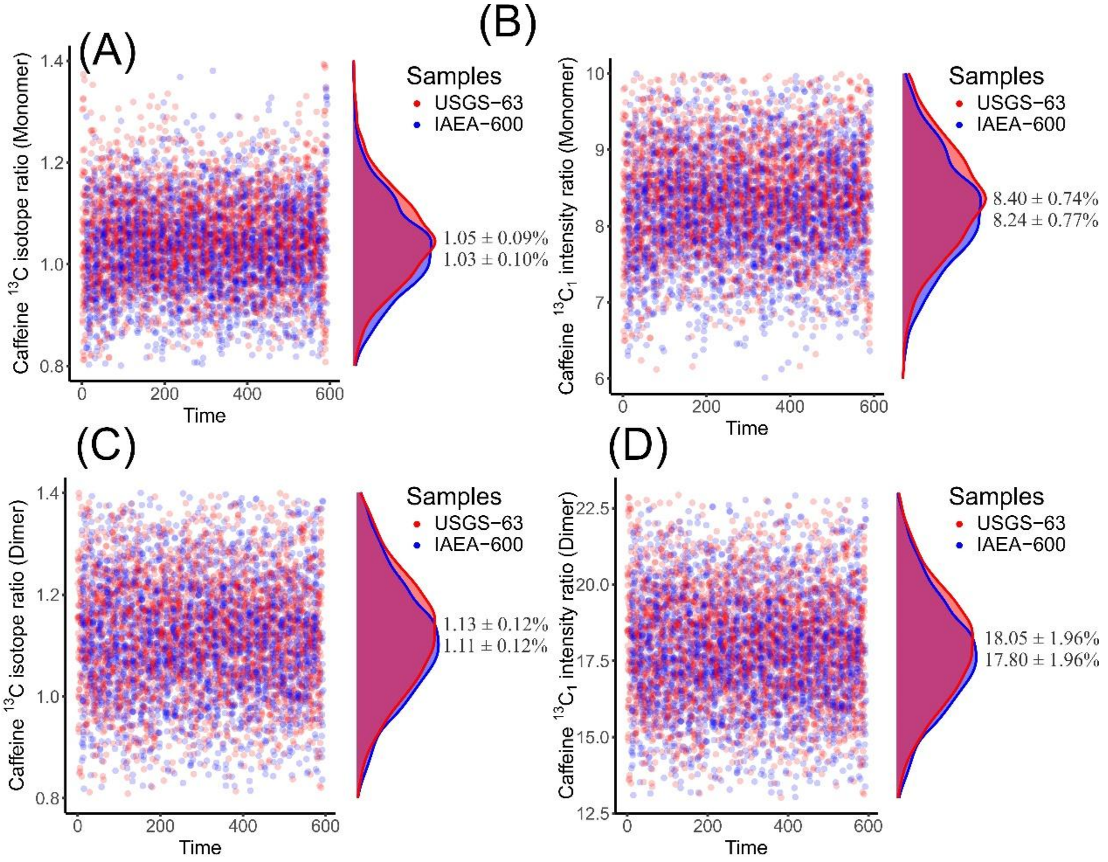

Fig. S3. Left: Isotope ratios ($^{13}C/^{12}C$) of caffeine standards detected as (A) Na-monomers and (C) Na-dimers. Values represent the intensity ratios of the A+1 ion over the monoisotopic ion in each mass spectrum (scatter plot), scaled to the number of each carbon atoms in the molecule (8 C atoms for the monomer and 16 C atoms for the dimer). **Right: Intensity ratios ($^{13}C_1/^{12}C_n$) of caffeine standards detected as (B) Na-monomers and (D) Na-dimers.** The dots indicate the intensity ratio measured over time by FT-ICR MS for USGS63 (red) and IAEA-600 (blue). Values represent the intensity ratios of the A+1 ion over the monoisotopic ion in each mass spectrum (scatter plot). Marginal histograms display the distribution of values, with mean and standard deviation annotated.

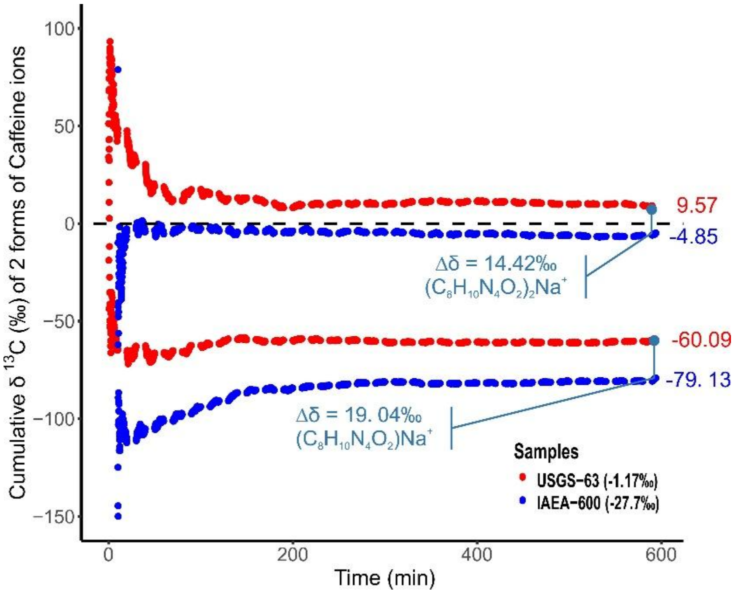

Fig. S4. Cumulative mean of δ^{13}C values over measurement time for the caffeine standards USGS63 (red) and IAEA-600 (blue) detected as Na-monomers (*n* = 5361) and Na-dimers (*n* = 5360). Data are presented as cumulative mean of spectra collected over time. The dashed line at 0‰ represents the reference material VPDB, corresponding to a $^{13}C/^{12}C$ isotope ratio of 1.11802%.

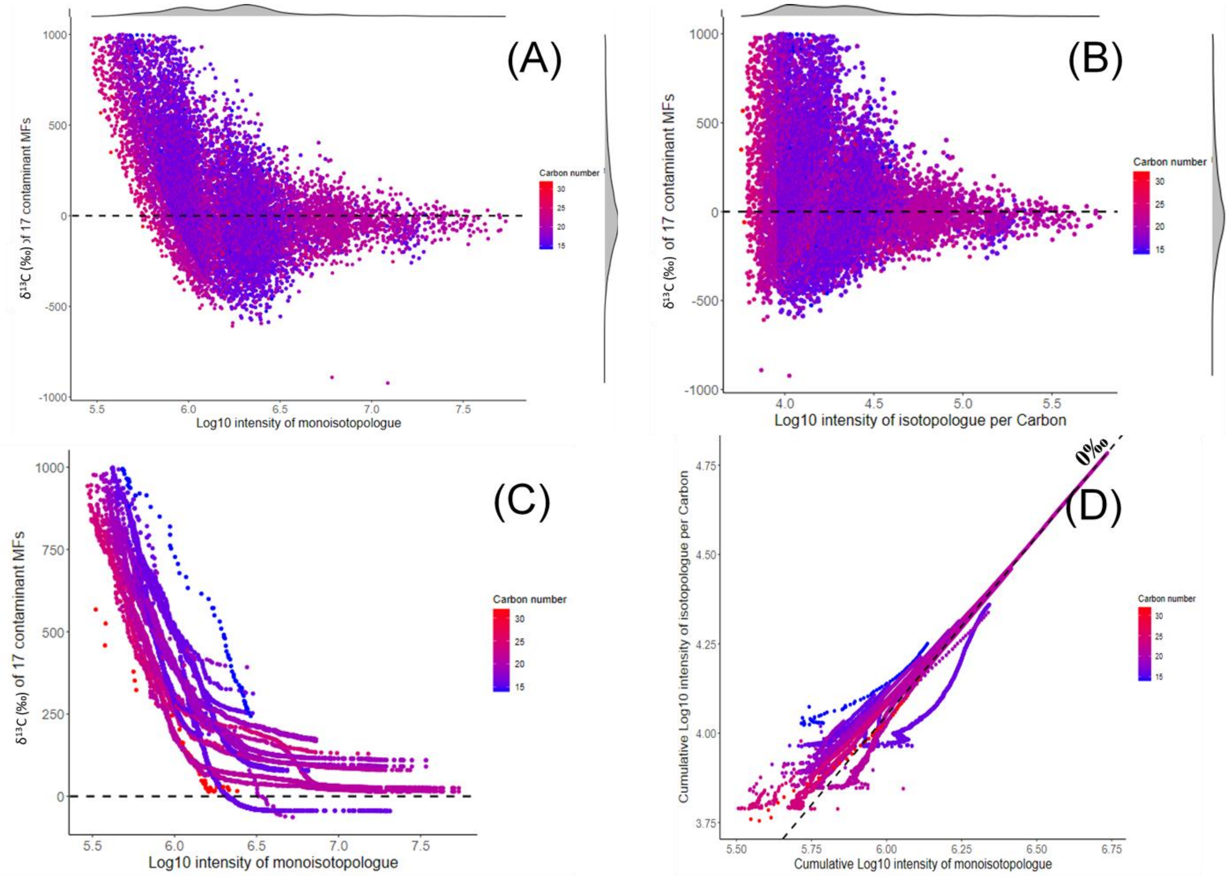

Fig. S5. Impact of FT-ICR MS peak intensity on the measured $\delta^{13}C$ values: (A) Measured $\delta^{13}C$ values of 19 molecular formulas (MFs) in water samples over the monoisotopologue intensity, (B) measured $\delta^{13}C$ values of 19 MFs in water samples over isotopologue intensity per carbon (*n* = 24,183), (C) cumulative means of measured $\delta^{13}C$ values of 19 MFs in water samples as cumulative Log10 intensity of monoisotopologues, (D) cumulative Log10 intensity of $^{13}C_1$ isotopologue per carbon for 19 MFs in water samples, plotted against the cumulative Log10 intensity of the corresponding monoisotopologues. Dashed line at 0‰ refers to the reference material VPDB ($^{13}C/^{12}C$ isotope ratio of 1.11802%). The intercepts of the regression lines represent the isotope ratios.

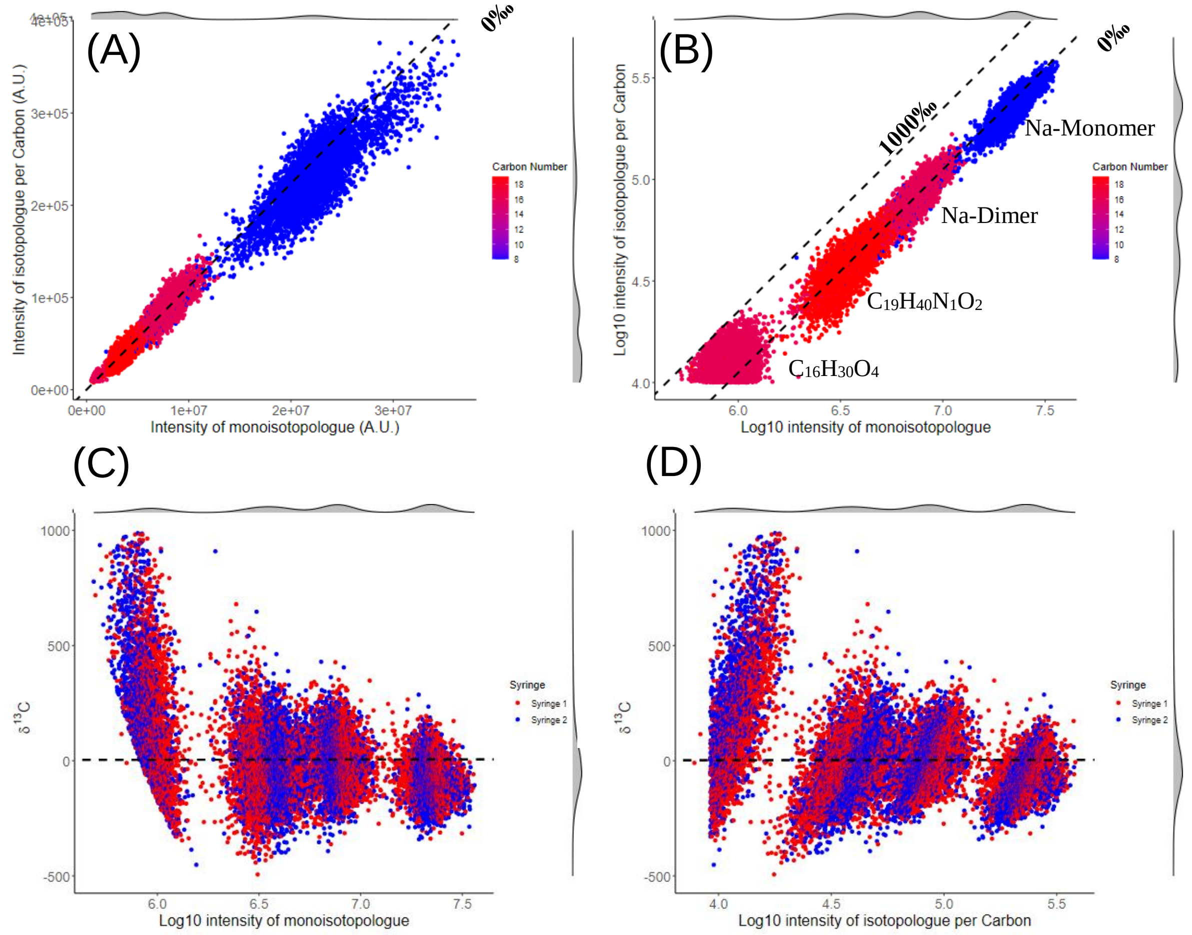

Fig. S6. Top: Linear response of the intensity of the $^{13}C_1$ isotopologue per carbon relative to the intensity of the monoisotopologues: (A) on arbitrary unit (A.U.), and (B) in a logarithmic scale. The black lines indicate the reference material VPDB with 0‰ ($^{13}C/^{12}C$ isotope ratio = 1.11802%, represented as y = 0.0111802×x in the raw scale (A) and y = x – 1.9503 in the logarithm scale (B)) and a cutoff line of 1000‰ for data processing, shown as y = x –1.65052 on the logarithmic scale. **Bottom: Relationship of $\delta^{13}C$ values and peak intensities:** (C) with isotopologues and (D) with $^{13}C_1$ isotopologue per carbon (n = 19,236) derived from four MFs (Na-monomer of caffeine, Na-dimer of caffeine, $C_{16}H_{30}O_4$ and $C_{19}H_{40}N_1O_2$, with numbers of averaged spectra of 5361, 5360, 3217 and 5298, respectively.) All $\delta^{13}C$ values were reported relative to the VPDB scale ($^{13}C/^{12}C$ isotope ratio ≡ 1.11802%).

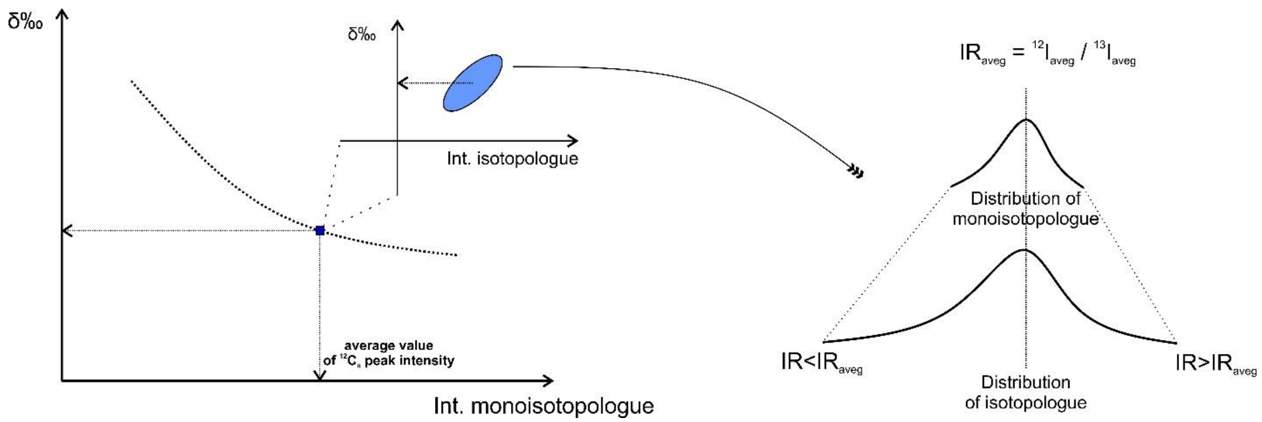

Fig. S7. Schematic of the effects of peak intensities on the measured δ^{13}C values. Isotope ratio (IR) is calculated from the mass peak magnitude of ^{12}C monoisotopologue (^{12}I) and $^{12}C_{n-1}{}^{13}C_1$ isotopologue (^{13}I). With thousands of consecutive spectra, the isotope ratio can be precisely determined (IR_{aveg}). The measured δ^{13}C values present an overall negative relationship as the ^{12}I increases, i.e. intensity-dependent variance. Within the same measurement, instrumental drift inevitably introduced fluctuations in peak intensities, and the measured δ^{13}C values showed a positive correlation with ^{13}I (insert). Because molecular formulas in NOM below 1000 Da typically have less than 50 C atoms, the ^{12}I is naturally higher than ^{13}I and shows a smaller standard deviation (s.d.) than that of ^{13}I, hence the s.d. of measured δ^{13}C values is governed by the s.d. of ^{13}I (right).

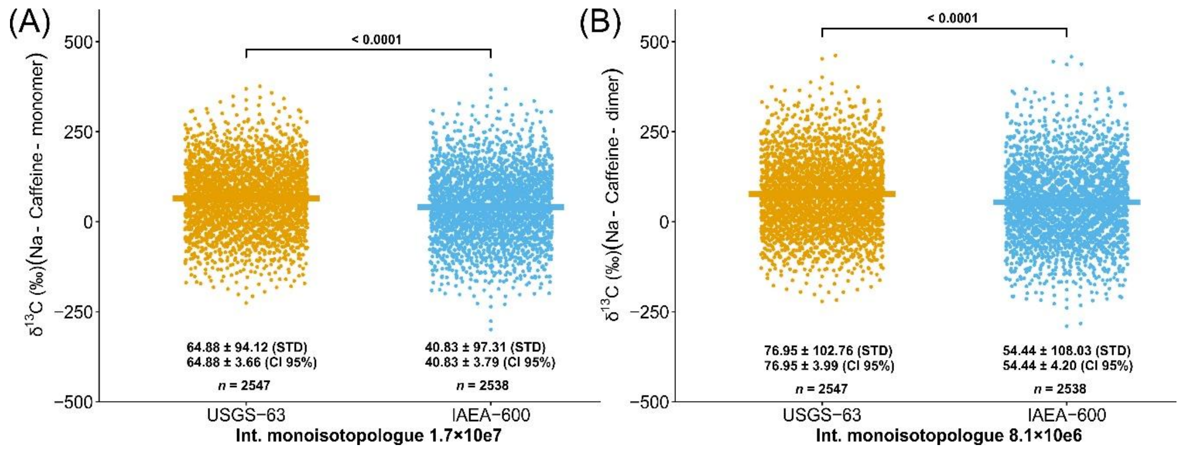

Fig. S8. $\delta^{13}C$ values for two caffeine standards using an intensity tuning: (A) Caffeine Na-monomer, with intensities of 1.76×10^7 (USGS63) and 1.72×10^7 (IAEA-600), and a relative error of 2.43% and (B) Caffeine Na-dimer, with intensities of 8.72×10^6 and 7.57×10^6, and a relative error of 14.15%. Statistical comparisons were performed using a two-sided t-test assuming equal variance. Error bars represent the 95% confidence intervals of the means. Comparable intensity of monoisotopologue is the precondition for the compound specific isotope analysis, i.e., obtaining $\Delta\delta^{13}C$ of a compound across samples.

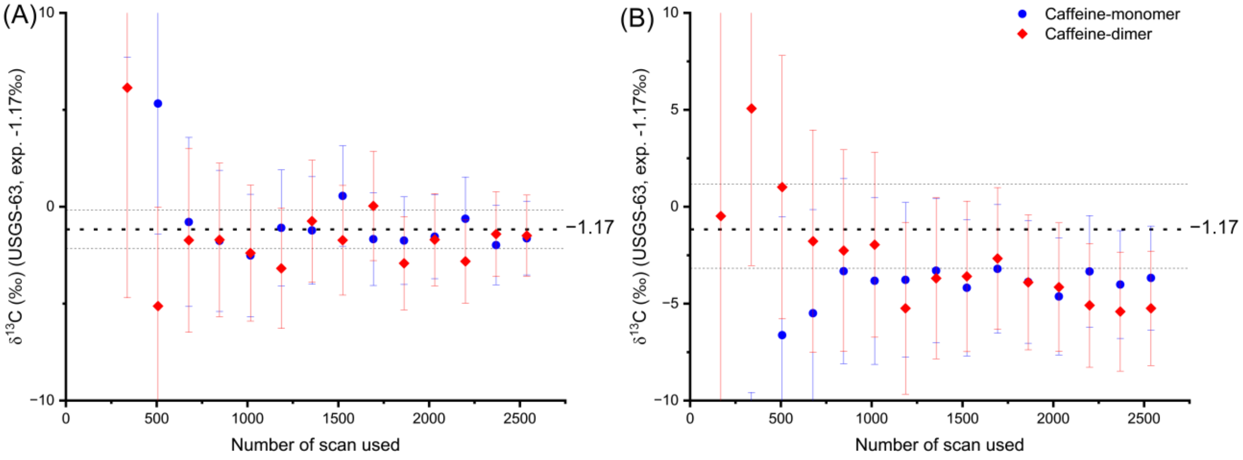

Fig. S9. Calibration of the caffeine monomer and dimer over the number of acquired spectra for each sample: (A) Two-point calibration - previously measured values were used to build a linear regression model to predict the $\delta^{13}C$ value of USGS63 using cumulative means obtained in subsequent intervals. The dashed gray lines indicate the 1‰ range. (B) One-point calibration - USGS63 was calibrated using the measured $\delta^{13}C$ values of IAEA-600. The dashed gray lines indicated the 2‰ range. Error bars represent the standard error of the measured $\Delta\delta^{13}C$ values.

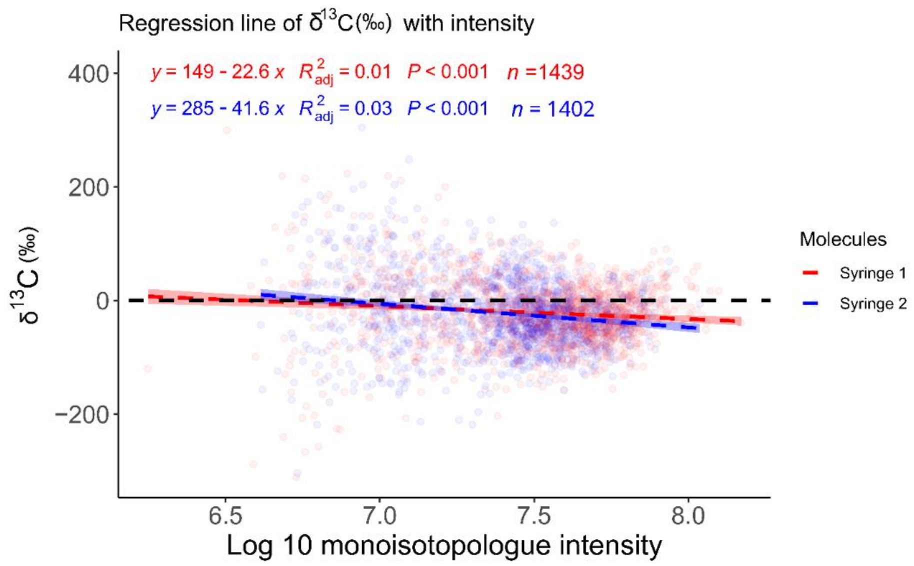

Fig. S10. FT-ICR MS derived isotope ratio measurements for oleic acid ($\delta^{13}C$ is expected at -29.7‰) with increasing flow rates and intensity of the monoisotopologues (n = 1439 and 1402 for syringe 1 and 2, respectively). Linear regression between measured $\delta^{13}C$ and Log10 intensity of the monoisotopologue, with ribbon representing the 95% confidence interval. Within overlapped intensity of the monoisotopologue of $10^{7.0}$-$10^{7.4}$ between two syringes, the $\delta^{13}C$ values were predicted at -9.2‰/-6.3‰ ($\Delta\delta^{13}C$ = -2.9‰) and -18.24‰/-22.84‰ ($\Delta\delta^{13}C$ = +4.6‰) for syringe 1 and 2 respectively.

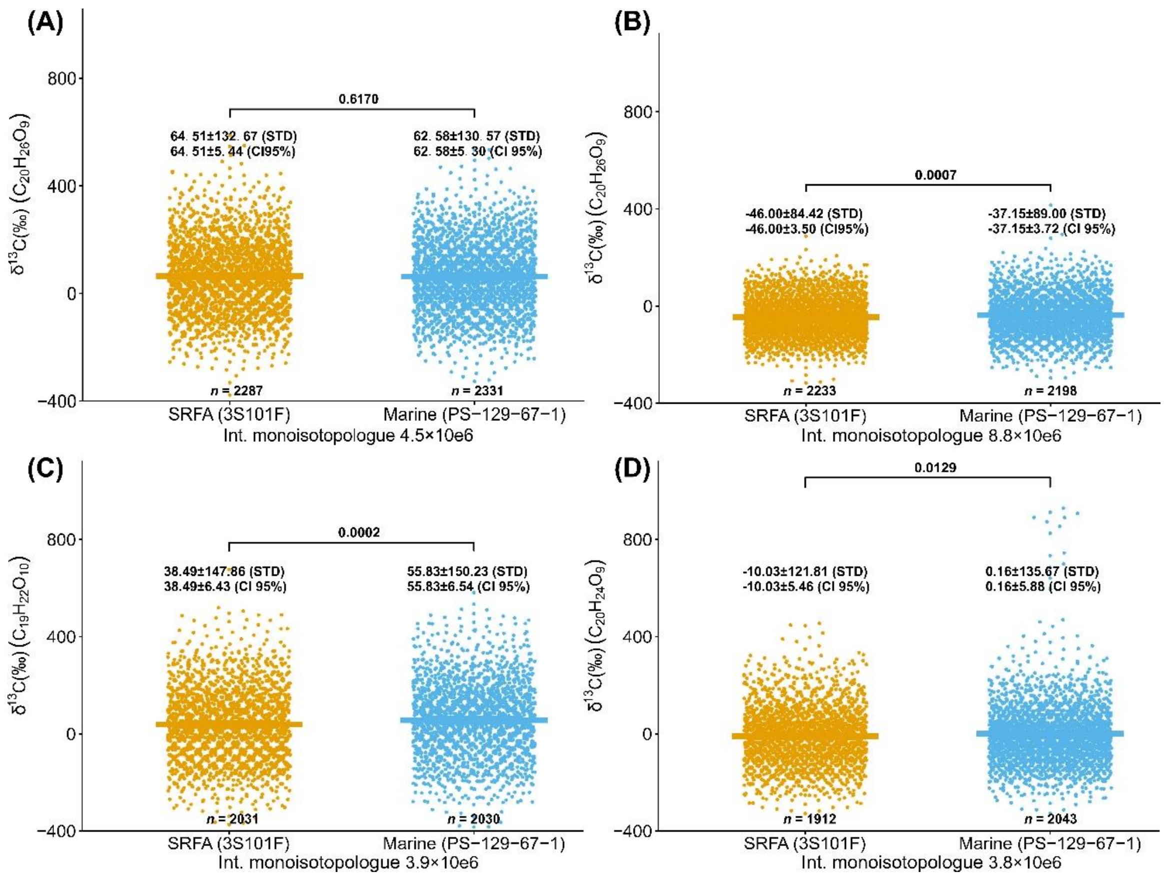

Fig. S11. Variance of δ^{13}C values of three marine molecular formulas present in SRFA and the marine sample: (A) $C_{20}H_{26}O_9$ measured at an averaged intensity of the monoisotopologue of 4.5×10^6 with a relative error of the monoisotopologue peak intensity of 1.63%; (B) $C_{20}H_{26}O_9$ measured at an averaged intensity of the monoisotopologue of 8.8×10^6 with a relative error of the averaged monoisotopologue peak intensity of 5.76%; (C) $C_{19}H_{22}O_{10}$ with a relative error of the averaged monoisotopologue peak intensity of 3.85% and (D) $C_{20}H_{24}O_9$ with a relative error of the averaged monoisotopologue peak intensity of 2.46%. Statistics were calculated with a 2-sided t-test, assuming equal variance. Error bars indicated confidence intervals (95%) of means.

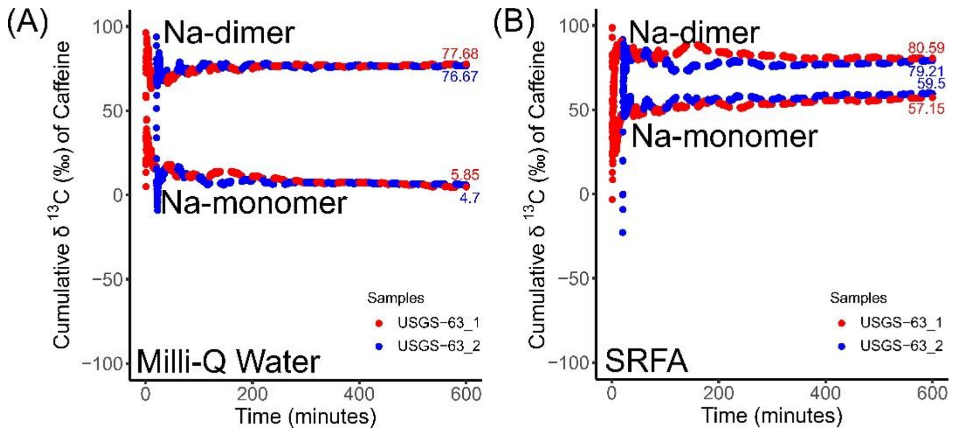

Fig. S12. Repeatability of $\delta^{13}C$ value measurements for caffeine (USGS63) in different matrices using intensity tuning strategy: (A) in ultrapure water with relative error of 0.98% and 1.03% in the averaged monoisotopologue peak intensity for the Na-monomer (*n* = 5358) and Na-dimer (**n** = 5358) respectively; (B) in 5 mg-C/L SRFA with a relative error of 2.62%, 4.74% in the averaged monoisotopologue peak intensity for the Na-monomer (*n* = 4999) and Na-dimer (*n* = 4999). Data were shown as the cumulative mean of spectra over time, and reported against reference material VPDB (^{13}C isotope ratio of 1.11802%, $\delta^{13}C$ of 0).

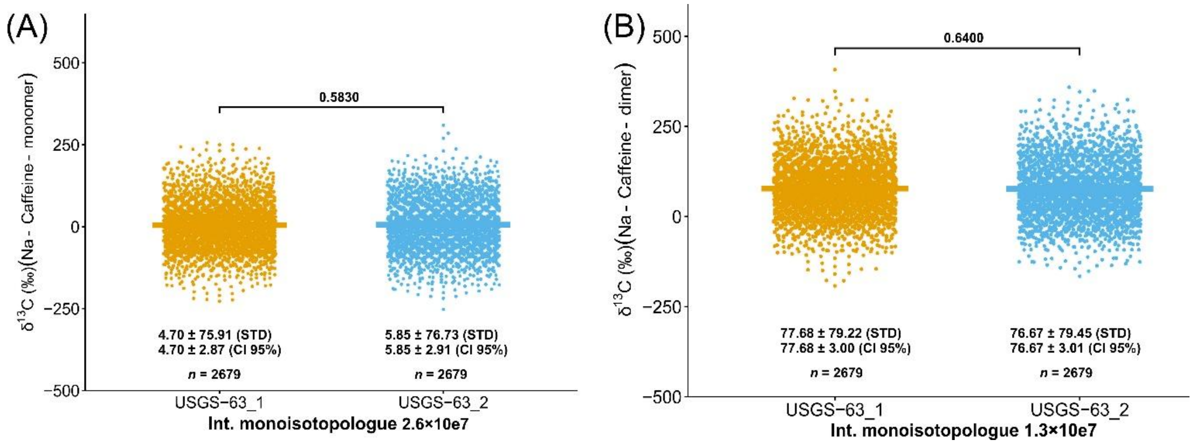

Fig. S13. Repeatability of $\delta^{13}C$ in ultrapure water for caffeine standards (USGS63) using intensity tuning strategy: (A) Caffeine Na-monomer, with a relative error of the averaged monoisotopologue peak intensity 0.98% and (B) Caffeine Na-dimer, with a relative error of the averaged monoisotopologue peak intensity of 1.03%. Statistics were calculated with a 2-sided t-test, assuming equal variance. Error bars indicate confidence intervals (95%) of means.

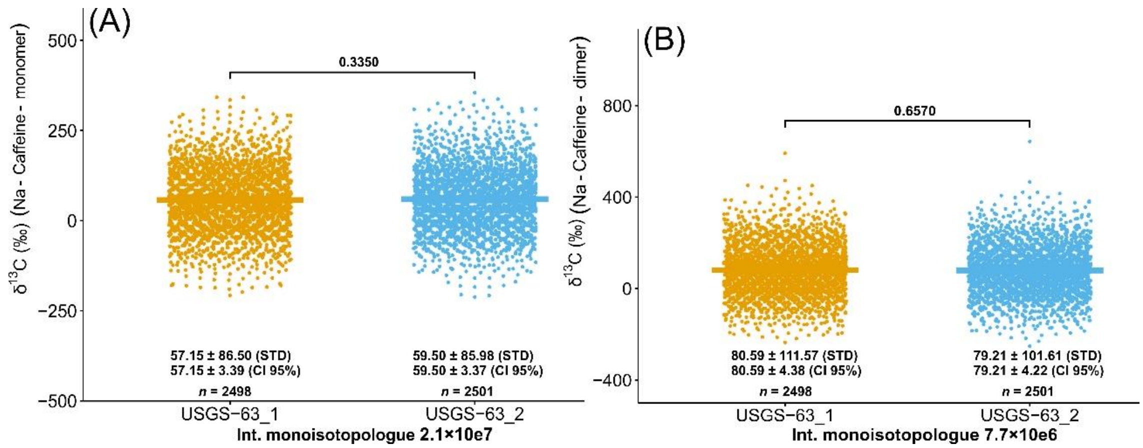

Fig. S14. Repeatability of $\delta^{13}C$ in SRFA for caffeine standards (USGS63) using the intensity tuning strategy: (A) Caffeine Na-monomer, with a relative error of the averaged monoisotopologue peak intensity of 2.62%; (B) Caffeine Na-dimer, with a relative error of the averaged monoisotopologue peak intensity of 4.74%. Statistics were calculated using a 2-sided t-test, assuming equal variance. Error bars indicate confidence intervals (95%) of means.

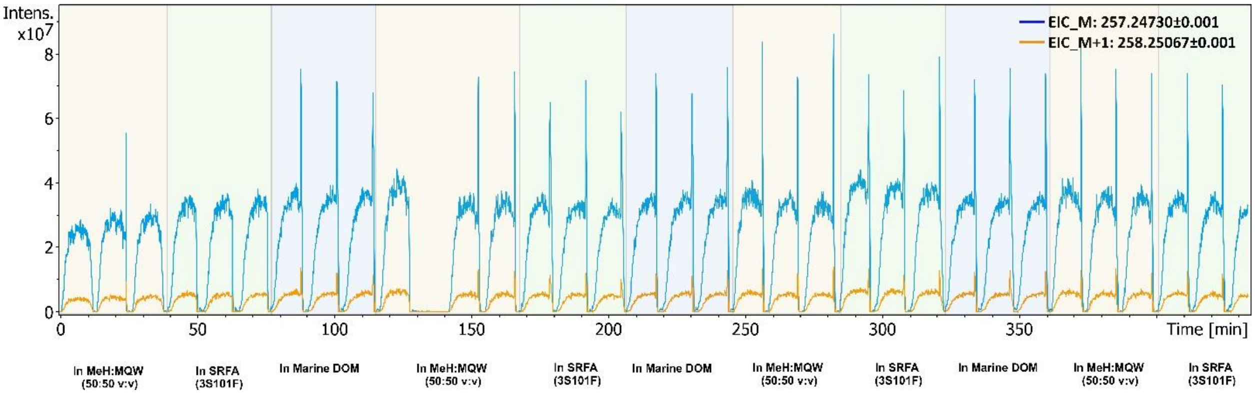

Fig. S15. Extracted ion chromatogram (EIC) of palmitic acid-d$_2$. The palmitic acid-d$_2$ was detected at m/z 257 as a sodium adduct, and the caffeine dimer was detected at m/z 411 as a sodium dimer. The total measurement lasted 433 minutes and consisted of 3858 spectra.

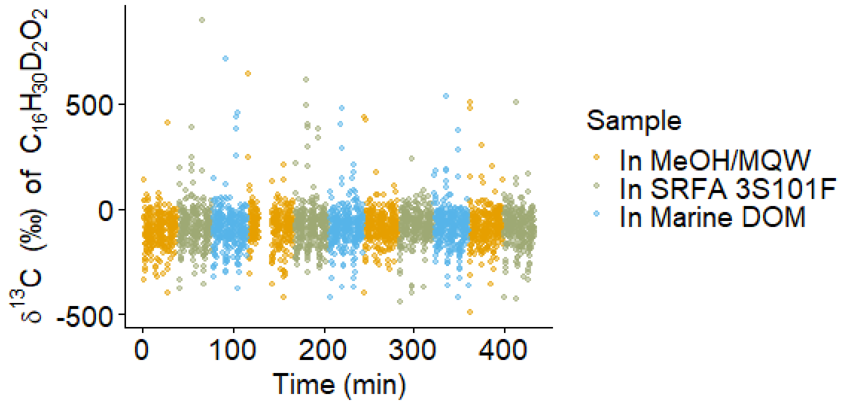

Fig. S16. The $\delta^{13}C$ values of palmitic acid-d$_2$ in different matrices. All $\delta^{13}C$ values including spectra with low mass peak abundance were included in the calculation of consecutive $\delta^{13}C$ means for analytes based on 1178 spectra for palmitic acid in MeOH/MWQ (ultrapure water), 1132 spectra in SRFA (3S101F), and 907 spectra in marine dissolved organic matter (DOM) were collected. Samples were infused with autosampler, and we excluded outliers with $^{12}C_n$ monoisotopologue peak intensities outside of 2 standard deviations of the averaged mean. After data trimming, 1086 spectra of palmitic acid in MeOH/MWQ (ultrapure water), 1035 spectra in SRFA, and 873 spectra in marine DOM were retained.

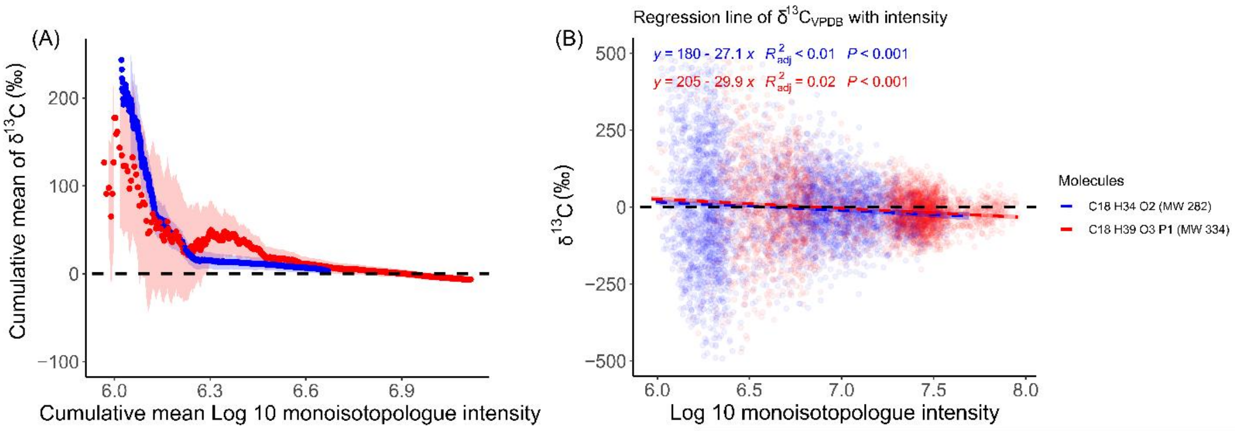

Fig. S17. Isotope ratio measurements for oleic acid ($\delta^{13}C$ is expected at -29.7‰) and octadecylphosphonic acid (ODPA, $\delta^{13}C$ is expected at -30.9‰): (A) Cumulative mean of the measured $\delta^{13}C$ values of oleic acid with increasing Log10 intensity of the monoisotopologue. At the same intensity ($10^{6.7}$), the $\delta^{13}C$ was measured at 3.59‰ (95% CI: -1.41‰ ~ 8.59‰) and 7.67‰ (95% CI: 0.9‰ ~14.44‰) for oleic acid and ODPA, respectively, and hence $\Delta\delta^{13}C$ was 5.28‰. No significant difference was found for the obtained $\delta^{13}C$ values (t-test, df = 3601.5, p = 0.3299). (B) Linear regression between measured $\delta^{13}C$ and Log10 intensity of the monoisotopologue, with ribbon representing the 95% confidence interval.

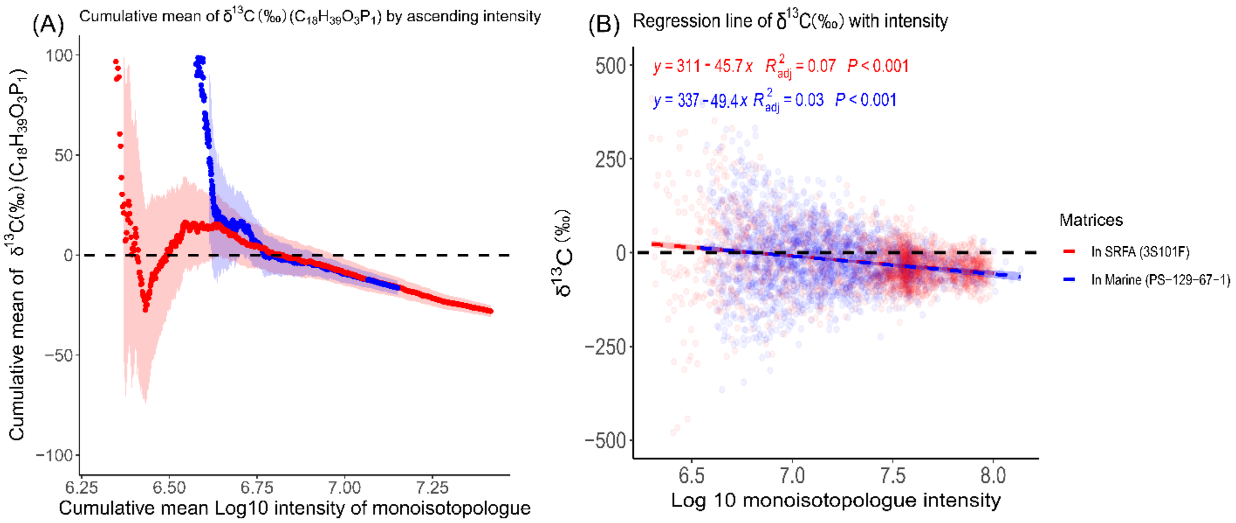

Fig. S18. Isotope ratio measurements for octadecylphosphonic acid (ODPA, δ^{13}C is expected at -30.9‰): (A) Cumulative mean of the measured δ^{13}C values of oleic acid with increasing Log10 intensity of the monoisotopologue. As the cumulative average mass peak magnitudes reached at 10^7 A.U., δ^{13}C was determined at -8.7‰ and -9.3‰ for ODPA in two syringes, corresponding to a standard deviation of 0.2‰, and a standard error of the mean of 0.2‰. (B) Linear regression between measured δ^{13}C and Log10 intensity of the monoisotopologue, with ribbon representing the 95% confidence interval (95% CI). The δ^{13}C was predicted at -9.5‰ (95% CI: -13.6‰ ~ -5.4‰) and -9.2‰ (95% CI: -13.0‰ ~ -5.3‰) for ODPA in two syringes at mass peak magnitudes of 10^7 A.U., corresponding to a standard deviation of 0.20‰, and a standard error of the mean of 0.15‰.

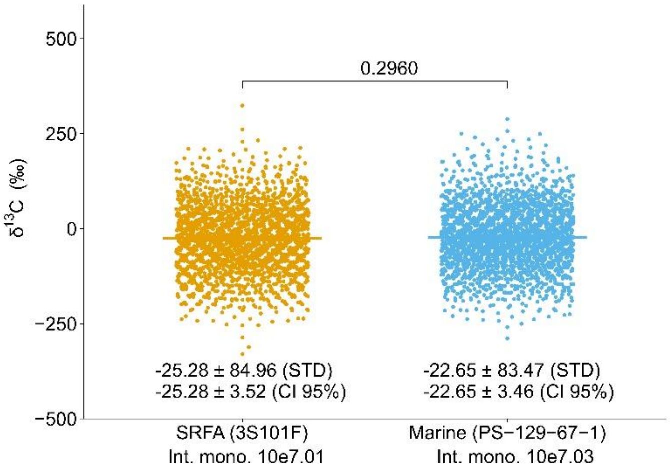

Fig. S19. Variance of δ^{13}C values for the molecular formula $C_{18}H_{22}O_6$ that was present in SRFA and the marine sample. Statistics were calculated using a 2-sided t-test, assuming equal variance. The δ^{13}C values of $C_{18}H_{22}O_6$ were measured at -25.3 ± 1.8‰ (s.e.) in SRFA and -22.7 ± 1.8‰ (s.e.) in the marine sample, yielding a Δδ^{13}C at 2.7 ± 2.5‰ (s.e.). Error bars indicate confidence intervals (95%) of means.

Table S1. Samples analyzed in this study with δ^{13}C values measured by IRMS.

Sample	Molecular formula	Molecular mass (Da)	δ^{13}C ‰
Caffeine (USGS63)	$C_8H_{10}N_4O_2$	194.080376	-1.17 ± 0.04*
Caffeine (IAEA-600)	$C_8H_{10}N_4O_2$	194.080376	-27.73 ± 0.04*
SRFA III (3S101F)	n.a.	n.a.	-28.2 ± 0.2
Marine (Weddell Sea surface)	n.a.	n.a.	-23.7 ± 0.1
Oleic acid STD	$C_{18}H_{34}O_2$	282.255880	-29.7 ± 0.2
Octadecylphosphonic acid (ODPA)	$C_{18}H_{39}O_3P$	334.263682	-30.9 ± 0.1
Palmitic acid - d_2	$C_{16}H_{30}D_2O_2$	258.252784	-31.2 ± 0.1

*Values and uncertainties reported in literature (*21*); n.a., not applicable.

Table S2. Description of samples and data acquisition.

Dataset	Samples	Concentration	Data acquisition	Comment
Caffeine_Int_differ	Caffeine	0.5 mg/L	ESI+, 4 M, 2ω, IAT 20 ms, ***full scan***.	
Int_match	Caffeine	0.5 mg/L	ESI+, 4 M, 2ω, IAT 50 ms, multi-***CASI***.	Peak intensities of caffeine were comparable among samples, indicating comparable ion populations.
	Oleic acid ($C_{18}H_{34}O_2$)	0.013 ng/L*	ESI-, 4M, 2ω, IAT 600 ms, ***CASI***.	
Caffeine_STD_matrix	Caffeine in SRFA	0.5 mg/L in 5 mg-C/L SRFA	ESI+, 4 M, 2ω, IAT 100 ms, multi-***CASI***.	Peak intensities of caffeine were comparable among samples, indicating comparable ion populations.
SRFA_Marine_marker	SRFA	5 mg-C/L	ESI-, 4 M, 2ω, IAT 600ms/400ms/200 ms, ***CASI***.	Molecules exhibited comparable intensities in both SRFA and marine samples. CASI centered at 409.61 Da, 409.65 Da and 407.63 Da for $C_{19}H_{22}O_{10}$, $C_{20}H_{26}O_9$, $C_{20}H_{24}O_9$.
	Marine	1 mg-C/L for $C_{19}H_{22}O_{10}$; 2 mg-C/L for $C_{20}H_{26}O_9$ and $C_{20}H_{24}O_9$		
C18_mass_differ	$C_{18}H_{34}O_2$ $C_{18}H_{39}O_3P$	0.13 ng/L*	ESI+, 4 M, 2ω, IAT 100 ms, multi-***CASI***.	Multi-CASI mode, with CASI centered at 281.75 Da and 333.76 Da for $C_{18}H_{34}O_2$ and $C_{18}H_{39}O_3P$.
NOM_calibration	SRFA	5 mg-C/L	ESI-, 4 M, 2ω, IAT 400 ms, multi-***CASI***.	Octadecylphosphonic acid ($C_{18}H_{39}O_3P$) was spiked as calibrant to 0.013 ng/L*. All molecular formulas exhibited comparable peak intensities across all samples. Multi-CASI centered at 333.63 Da for $C_{18}H_{22}O_6$ and 333.76 Da for ODPA.
	Marine	10 mg-C/L		
Palmitic_acid_matrices_LC	Palmitic acid-d_2	0.06 mg/L in MeOH:MQW (50:50 v:v)	ESI-, 4 M, 2ω, IAT 30 ms, ***CASI***.	Peak intensities of palmitic acid - d2 were comparable among samples, indicating comparable ion populations. CASI centered at 257.75 Da for palmitic acid-d_2.
	Palmitic acid-d_2	1 mg/L in 5 mg-C/L SRFA		
	Palmitic acid-d_2	1.5 mg/L in 10.9 mg-C/L Marine DOM		

*: Since dilution and flow rates changed, the concentrations here are estimated and not accurate.

Table S3. Peaks with a prominent $^{13}C_1$ isotopologue observed in the Caffeine_Int_differ dataset.

No.	Observed m/z	Molecular formula	Comments
1	195.087652	$[C_8H_{10}N_4O_2]H^+$	H-Caffeine monomer
1	217.069596	$C_8H_{10}N_4O_2Na_1^+$	Na-Caffeine monomer
2	265.119901	$C_{16}H_{18}O_2Na_1^+$	
3	279.229451	$C_{16}H_{32}O_2Na_1^+$	
4	281.172330	$C_{14}H_{26}O_4Na_1^+$	
5	287.281881	$C_{17}H_{37}N_1O_2^+$	
6	301.141030	$C_{16}H_{22}O_4Na_1^+$	
7	307.260751	$C_{18}H_{36}O_2Na_1^+$	
8	309.203630	$C_{16}H_{30}O_4Na_1^+$	
9	315.313181	$C_{19}H_{41}N_1O_2^+$	
10	317.114815	$C_{19}H_{18}O_3Na_1^+$	
11	353.266230	$C_{19}H_{38}O_4Na_1^+$	
12	381.297520	$C_{21}H_{42}O_4Na_1^+$	
13	393.297531	$C_{22}H_{42}O_4Na_1^+$	
14	397.271316	$C_{24}H_{38}O_3Na_1^+$	
15	411.149972	$C_{16}H_{20}N_8O_4Na_1^+$	Na-Caffeine dimer
16	413.266230	$C_{24}H_{38}O_4Na_1^+$	
17	481.313575	$C_{25}H_{46}O_7Na_1^+$	
18	485.381260	$C_{26}H_{54}O_6Na_1^+$	
19	579.292839	$C_{32}H_{44}O_8Na_1^+$	
